# Exosomal circRHCG promotes breast cancer metastasis via facilitating M2 polarization through TFEB ubiquitination and degradation

**DOI:** 10.1038/s41698-024-00507-y

**Published:** 2024-01-29

**Authors:** Hong-yu Shen, Jia-lin Xu, Wei Zhang, Qin-nan Chen, Zhen Zhu, Yuan Mao

**Affiliations:** 1 Gusu School, The Affiliated Suzhou Hospital of Nanjing Medical University, Nanjing Medical University, Suzhou, China; 2 Department of General Surgery, The First Affiliated Hospital of Nanjing Medical University, Nanjing, China; 3 Division of Gastrointestinal Surgery, The Affiliated Huai’an Hospital of Xuzhou Medical University, Huai’an, China; 4 Department of Clinical Medicine, Jiangsu Health Vocational College, Nanjing, China; 5 Department of Oncology, The Fourth Affiliated Hospital of Nanjing Medical University, Nanjing, China

**Keywords:** Breast cancer, Breast cancer

## Abstract

Triple-negative breast cancer (TNBC) is a highly aggressive cancer with distant metastasis. Accumulated evidence has demonstrated that exosomes are involved in TNBC metastasis. Elucidating the mechanism underlying TNBC metastasis has important clinical significance. In the present study, exosomes were isolated from clinical specimens and TNBC cell lines. Colony formation, EdU incorporation, wound healing, and transwell assays were performed to examine TNBC cell proliferation, migration, and metastasis. Macrophage polarization was evaluated by flow cytometry and RT-qPCR analysis of polarization markers. A mouse model of subcutaneous tumor was established for assessment of tumor growth and metastasis. RNA pull-down, RIP and Co-IP assays were used for analyzing molecular interactions. Here, we proved that high abundance of circRHCG was observed in exosomes derived from TNBC patients, and increased exosomal circRHCG indicated poor prognosis. Silencing of circRHCG suppressed TNBC cell proliferation, migration, and metastasis. TNBC cell-derived exosomes promoted M2 polarization via delivering circRHCG. Exosomal circRHCG stabilized BTRC mRNA via binding FUS and naturally enhanced BTRC expression, thus promoting the ubiquitination and degradation of TFEB in THP-1 cells. In addition, knockdown of BTRC or overexpression of TFEB counteracted exosomal circRHCG-mediated facilitation of M2 polarization. Furthermore, exosomal circRHCG promoted TNBC cell proliferation and metastasis by facilitating M2 polarization. Knockdown of circRHCG reduced tumor growth, metastasis, and M2 polarization through the BTRC/TFEB axis in vivo. In summary, exosomal circRHCG promotes M2 polarization by stabilizing BTRC and promoting TFEB degradation, thereby accelerating TNBC metastasis and growth. Our study provides promising therapeutic strategies against TNBC.

## Introduction

Triple-negative breast cancer (TNBC) is characterized by negative expression of estrogen receptor, progesterone receptor and human epidermal growth factor receptor 2^1^, accounting for roughly 15% of all cases^2^. TNBC is the most aggressive breast cancer that grows and spreads fast, causing increased risk of recurrence, high metastasis, and a bad outcome^3^. Due to the triple-negative phenotype, the treatment for TNBC is limited, and patients cannot benefit from therapies against these receptors. Even so, the prognosis has been greatly improved in recent years thanks to advances in treatment including surgery, chemotherapy, radiation therapy, and immunotherapy^4,5^. However, exploring the mechanism underlying TNBC metastasis is crucial for understanding TNBC pathogenesis and developing promising therapeutic strategies.

Exosomes are nanosized cell-derived vesicles of ~30–200 nm in diameter that contain abundant cellular constituents such as nucleic acids, serving key roles in intercellular communication and regulating the behavior and activity of recipient cells^6,7^. Intriguingly, growing evidence has shown that exosomes are important regulators of TNBC. A recent study reported that elevated exosomal annexin A2 in the serum of TNBC patients might enhance angiogenesis^8^. Li et al. found that exosomal cluster of differentiation 151 (CD151) promoted TNBC migration and invasion^9^. Additionally, exosomes are implicated in the regulation of drug resistance in TNBC^10^. Thus, exosomes are becoming a promising endogenous vehicle for drug delivery to improve anti-tumor activities in TNBC. For example, an in vitro study showed that exosomes released by mesenchymal stem cells suppressed aggressiveness in TNBC via delivering microRNA-381-3p (miR-381-3p)^11^. Tumor-released exosomes are key regulators in macrophage polarization. However, exosome-mediated crosstalk between tumor-associated macrophages and tumor cells remains poorly understood.

Circular RNAs (circRNAs) regulate target gene expression and are emerging as vital regulators in cancers. CircRNAs regulate the polarization of tumor-associated macrophages and serve significant roles in tumor metastasis and progression^12^. Although several circRNAs have been reported to act as oncogenes to promote TNBC progression^13,14^, the roles of exosomal circRNA in TNBC need better understanding. Specially, circRNAs are enriched in exosomes and exosomal circRNAs modulate the growth, invasion, and metastasis in cancers including in TNBC^15^. Yang and colleagues found that exosomal circPSMA1 promoted the tumorigenesis and metastasis in TNBC^16^. CircRHCG, termed as hsa_circ_ 0104852, is highly abundant in exosomes derived from TNBC cell line MDA-MB-231^17^. However, the role of exosomal circRHCG in TNBC is still unknown.

Transcription Factor EB (TFEB) is a key transcription factor that controls lysosomal biogenesis and autophagy^18^. Importantly, increased TFEB expression is connected to the aggressiveness of breast cancer^19^. Conversely, knockout of TFEB in macrophages enhanced breast cancer growth via promoting M2 polarization^20^. Thus, elucidating the roles of TFEB in tumor cells and tumor-associated macrophages is important. As exosomes play important roles in shaping tumor microenvironment, we hypothesized that TNBC cell-derived exosomes might regulate macrophage polarization via delivering circRHCG and adjusting TFEB expression, thus regulating TNBC progression.

In summary, we sought to explore the activity of exosomal circRHCG in regulating macrophage and TNBC cell metastasis. In this study, we demonstrated that exosomal circRHCG facilitated M2 polarization by enhancing β-transducin repeat containing E3 ubiquitin protein ligase (BTRC)-dependent ubiquitination and degradation of TFEB, thus promoting cancer cell proliferation, migration, invasion, and metastasis in TNBC. Our study provides a mechanistic insight into exosome-mediated regulation of tumor microenvironment and metastasis in TNBC and suggests potential therapeutic targets and strategies for TNBC.

## Results

### Increased exosomal circRHCG was associated with poor prognosis in TNBC patients

**Fig. 1:**
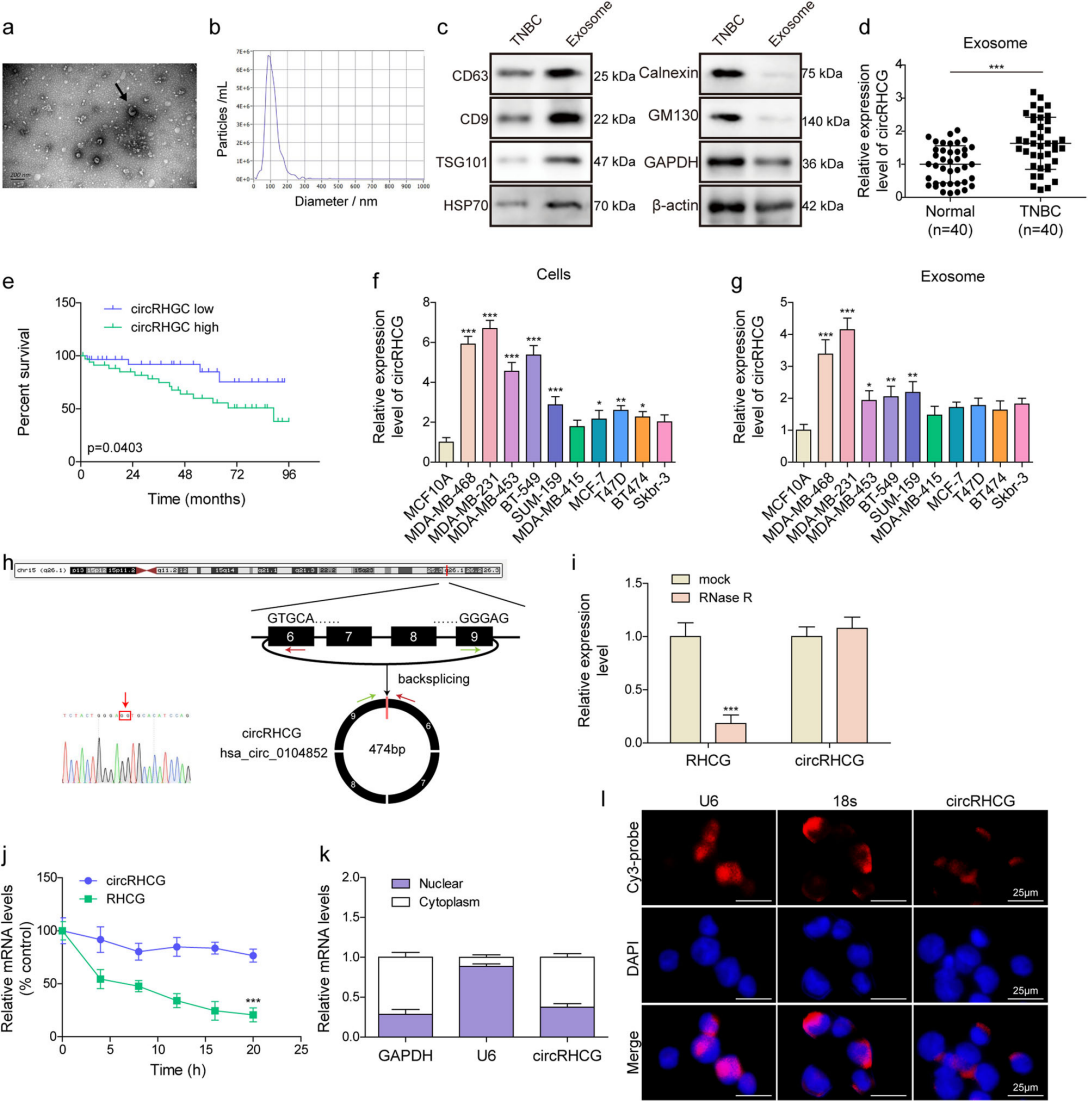
Increased exosomal circRHCG was associated with poor prognosis. **a** TEM examination of exosomes. **b** NTA analysis of exosome size. **c** Western blotting analysis of CD63, CD9, TSG101, HSP70, Calnexin, GM130, GAPDH and β-actin in TNBC cells and exosomes. **d** RT-qPCR analysis of circRHCG in exosomes derived from volunteers (*n* = 40) and TNBC patients (*n* = 40). **e** Overall survival of patients (circRHCG high, *n* = 20; circRHCG low, *n* = 20). **f**, **g** RT-qPCR analysis of circRHCG in breast cancer cell lines and exosomes derived from these cell lines (*n* = 3). **h** Genomic loci of circRHCG and its back-splicing site. **i**, **j** RT-qPCR analysis of RHCG mRNA and circRHCG after RNase R digestion and actinomycin D treatment (*n* = 3). **k** RNA levels of GAPDH, U6 and circRHCG in the nuclear and cytosolic fractions (*n* = 3). **l** FISH assays for analysis of circRHCG localization with Cy3-conjugated probes (Red). DAPI (Blue) was used for nuclear staining. Scale bar, 25 µm. *p < 0.05, **p < 0.01 and ***p < 0.001. Error bars indicate SD. All results are representative of or combined from at least three independent experiments.

Exosomes were isolated from blood samples of TNBC patients and volunteers, normal human mammary epithelial MCF10A cells and TNBC cells including MDA-MB-231, BT-549, MDA-MB-468, SUM-159, and MDA-MB-453. Typical vehicles of 30-150 nm in diameter were confirmed by TEM and NTA (Fig. 1a, b), respectively. High expression of exosome markers including CD9, CD63, TSG101, and HSP70 was observed in exosomes, but not in TNBC cells (Fig. 1c). Negative exosome markers Calnexin and GM130 was abundant in TNBC cells, but not in exosomes (Fig. 1c). Ponceau staining was used to confirm that equal amount of proteins from exosomes and TNBC cells were loaded (Supplementary Fig. 1). These observations validated that exosomes were successfully isolated. TNBC patient-derived exosomes showed high levels of circRHCG compared to exosomes from volunteers (Fig. 1d). Besides, TNBC patients with high exosomal circRHCG levels exhibited poor survival (Fig. 1e). Compared to non-tumorigenic epithelial MCF-10A cells, non-TNBC cell lines MCF-7, T47D and BT474 showed moderately increased circRHCG expression, and no significant change was observed in MDA-MB-415 and Skbr-3 cells and exosomes from these non-TNBC cell lines (Fig. 1f, g). However, circRHCG was highly upregulated in TNBC cell lines including MDA-MB-231, BT-549, MDA-MB-468, SUM-159 and MDA-MB-453 and exosomes from these TNBC cell lines (Fig. 1f, g). MDA-MB-468 and MDA-MB-231 cells were chosen for subsequent assays as they showed highest levels of circRHCG. As shown in Fig. 1h, circRHCG (hsa_circ_0104852) is generated from exon 6 to 9 of *RHCG* gene via back-splicing with a length of 474 bp, and the back-splicing site was verified by Sanger sequencing. As expected, compared to linear RHCG mRNA, circRHCG was highly resistant to RNase R treatment and showed longer half-time after actinomycin D treatment, demonstrating high stability of the circular structure of circRHCG (Fig. 1i, j). Furthermore, we observed that circRHCG was mainly enriched in the cytoplasm, which was validated by fluorescence in situ hybridization (FISH) assays (Fig. 1k, l). These observations suggested high abundance of exosomal circRHCG in TNBC cells and patients.

### Knockdown of circRHCG restrained TNBC cell proliferation, migration, invasion and EMT

**Fig. 2:**
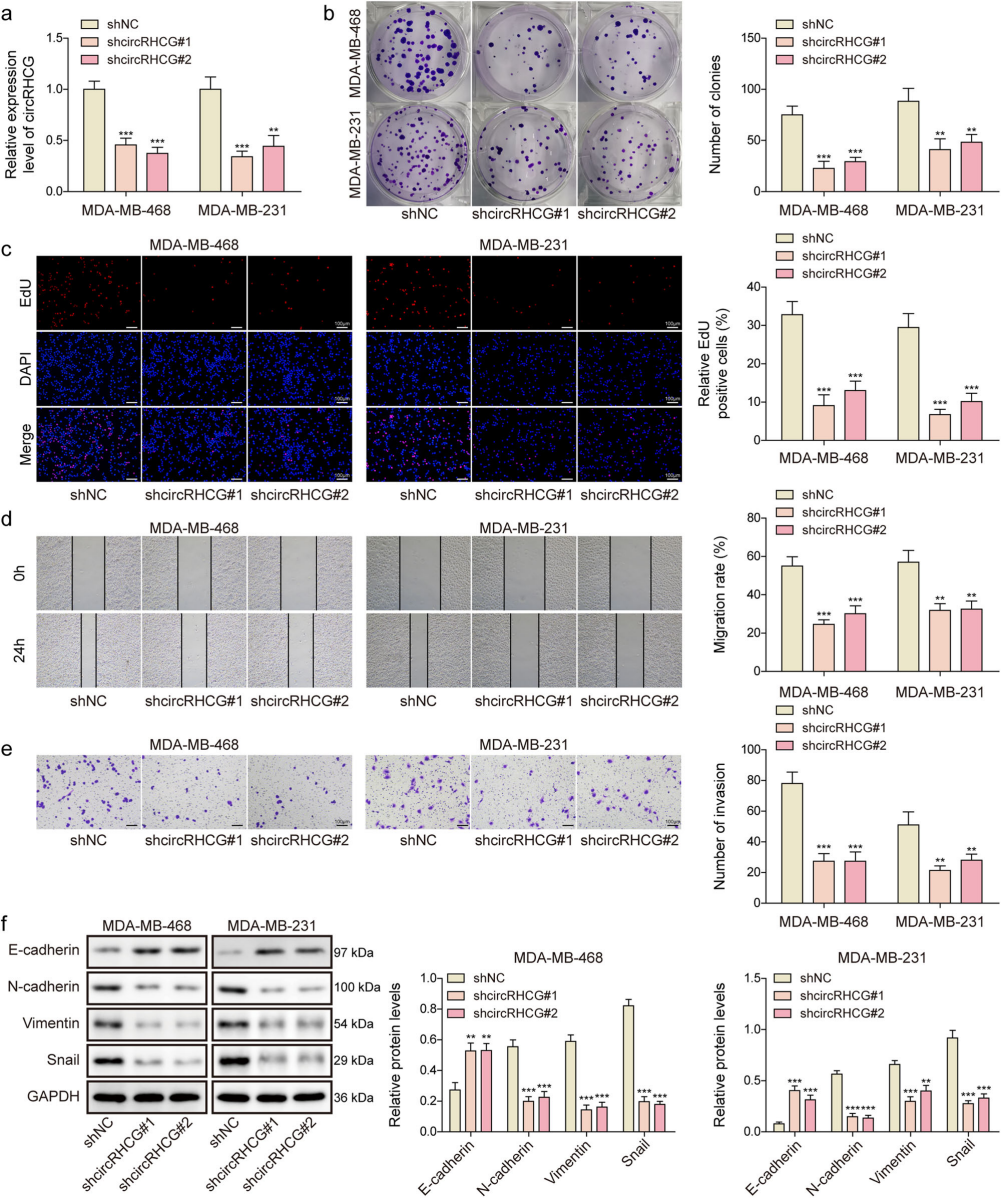
Knockdown of circRHCG restrained TNBC cell proliferation, migration, invasion and EMT. CircRHCG was knocked down in MDA-MB-468 and MDA-MB-231 cells through shRNA transfection. **a** RT-qPCR analysis of circRHCG (*n* = 3). **b** Colony formation analysis (*n* = 3). **c** EdU (Red) incorporation assays for evaluating cell proliferation (*n* = 3). The nuclei were stained with DAPI (Blue). Scale bar, 100 µm. **d** Wound healing assays for cell migration analysis (*n* = 3). **e** Tanswell assays for analyzing cell invasion (*n* = 3). **f** Protein levels of E-cadherin, N-cadherin, Vimentin, Snail an GAPDH (*n* = 3). **p < 0.01 and ***p < 0.001. Error bars indicate SD. All results are representative of or combined from at least three independent experiments.

CircRHCG was knocked down through shcircRHCG transfection, which was confirmed by RT-qPCR (Fig. 2a). Obviously, the colony forming capacity of MDA-MB-468 and MDA-MB-231 cells was impaired by silencing of circRHCG (Fig. 2b). Low EdU incorporation in circRHCG-knockdown cells suggested that knockdown of circRHCG reduced cell proliferation (Fig. 2c). Moreover, silencing of circRHCG significantly restrained TNBC cell migration and invasion (Fig. 2d, e). Furthermore, we found increased E-cadherin expression but decreased expression of N-cadherin, Vimentin, and Snail in circRHCG-silencing cells, suggesting suppression of EMT in these cells (Fig. 2f). These findings indicated that circRHCG might promote TNBC growth and metastasis.

### TNBC cell-derived exosome promoted M2 polarization dependent on circRHCG

**Fig. 3:**
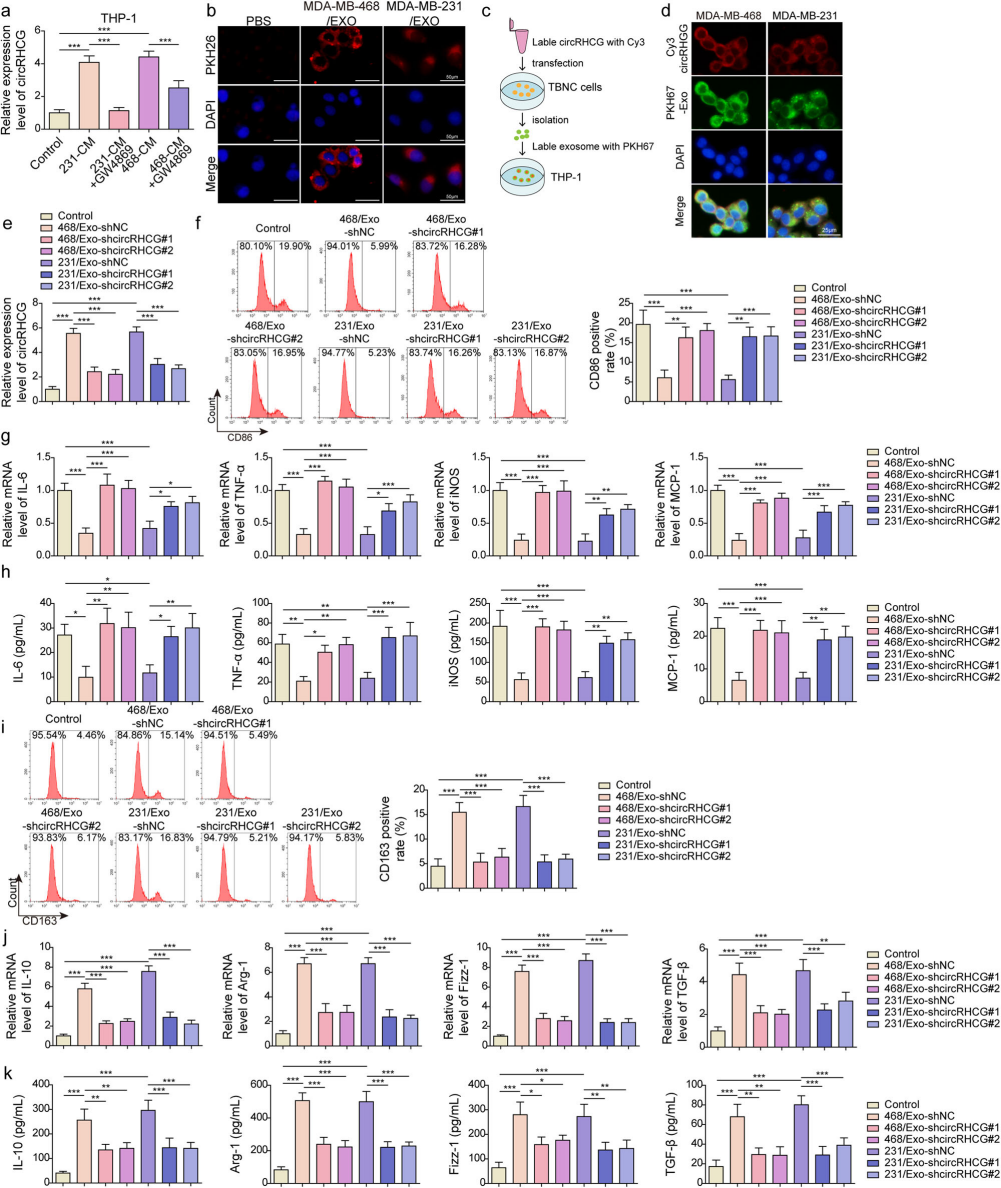
TNBC cell-derived exosome promoted M2 polarization dependent on circRHCG. **a** RT-qPCR analysis of circRHCG in THP-1 cells treated with MDA-MB-468-CM and MDA-MB-231-CM (*n* = 3). GW4869 was used to block exosome generation. **b** The entry of exosomes into THP-1 cells. Exosomes were isolated from MDA-MB-468 and MDA-MB-231 cells transfected with shNC, shcircRHCG#1 or shcircRHCG#2 and co-cultured with THP-1 cells pre-treated with PMA. **c** Cy3-labeled circRHCG was transfected into MDA-MB-468 and MDA-MB-231, and exosomes were extracted and labeled with PKH67. THP-1 cells were incubated with exosomes. **d** Colocalization of circRHCG (Red) and PKH67-labeled exosomes (green) was imaged via fluorescence microscopy. **e** RT-qPCR analysis of circRHCG (*n* = 3). **f** CD86-positive THP-1 cells were examined by flow cytometry (*n* = 3). **g** RT-qPCR analysis of IL-6, TNF-α, iNOS and MCP-1 in THP-1 cells (*n* = 3). **h** The concentration of IL-6, TNF-α, iNOS, and MCP-1 was determined with ELISA (*n* = 3). **i** CD163-positive THP-1 cells were examined by flow cytometry (*n* = 3). **j** RT-qPCR analysis of IL-10, Arg-1, Fizz-1, and TGF-β in THP-1 cells (*n* = 3). **k** The concentration of IL-10, Arg-1, Fizz-1, and TGF-β was determined with ELISA (*n* = 3). **p < 0.01 and ***p < 0.001. Error bars indicate SD. All results are representative of or combined from at least three independent experiments.

THP-1 cells were maintained in MDA-MB-468-conditioned media (468-CM) and MDA-MB-231-conditioned media (231-CM), and we observed that THP-1 cells showed increased circRHCG expression (Fig. 3a). However, the pre-treatment of GW4869, an inhibitor of exosome generation, abolished CM-mediated upregulation of circRHCG, suggesting that exosomes in CM delivered circRHCG into THP-1 cells (Fig. 3a). Indeed, PKH26-labeled exosomes could enter THP-1 cells, and no PKH26 fluorescence was detected in THP-1 cells treated with an equal volume of the PKH26-PBS control (Fig. 3b). To further verify the internalization of exosomes, Cy3-labeled circRHCG was transfected into MDA-MB-468 and MDA-MB-231, and exosomes were extracted and labeled with PKH67. THP-1 cells were incubated with exosomes (Fig. 3c). Co-localization of circRHCG and PKH67-labeled exosomes were observed in THP-1 cells (Fig. 3d), revealing that circRHCG exosomes could be efficiently taken up by THP-1 cells. THP-1 cells were co-cultured with MDA-MB-468 and MDA-MB-231-derived exosomes, and circRHCG expression was enhanced (Fig. 3e). CircRHCG-silencing exosomes failed to upregulate circRHCG (Fig. 3e). Subsequently, we examined the effect of exosomal circRHCG on THP-1 polarization. MDA-MB-468 and MDA-MB-231 -derived exosomes reduced CD86-positive M1 cells and reduced the expression and secretion of M1 markers IL-6, TNF-α, iNOS and MCP-1, which were counteracted by exosomal knockdown of circRHCG (Fig. 3f–h). Conversely, MDA-MB-468 and MDA-MB-231 -derived exosomes raised CD163-positive M2 cells and promoted the expression and secretion of M2 markers IL-10, Arg-1, Fizz-1, and TGF-β, and exosomal knockdown of circRHCG abolished these effects (Fig. 3i–k). Collectively, our findings implied that TNBC cell-derived exosome promoted M2 polarization via delivering circRHCG.

### Exosomal circRHCG enhanced the ubiquitination and degradation of TFEB via upregulating BTRC

**Fig. 4:**
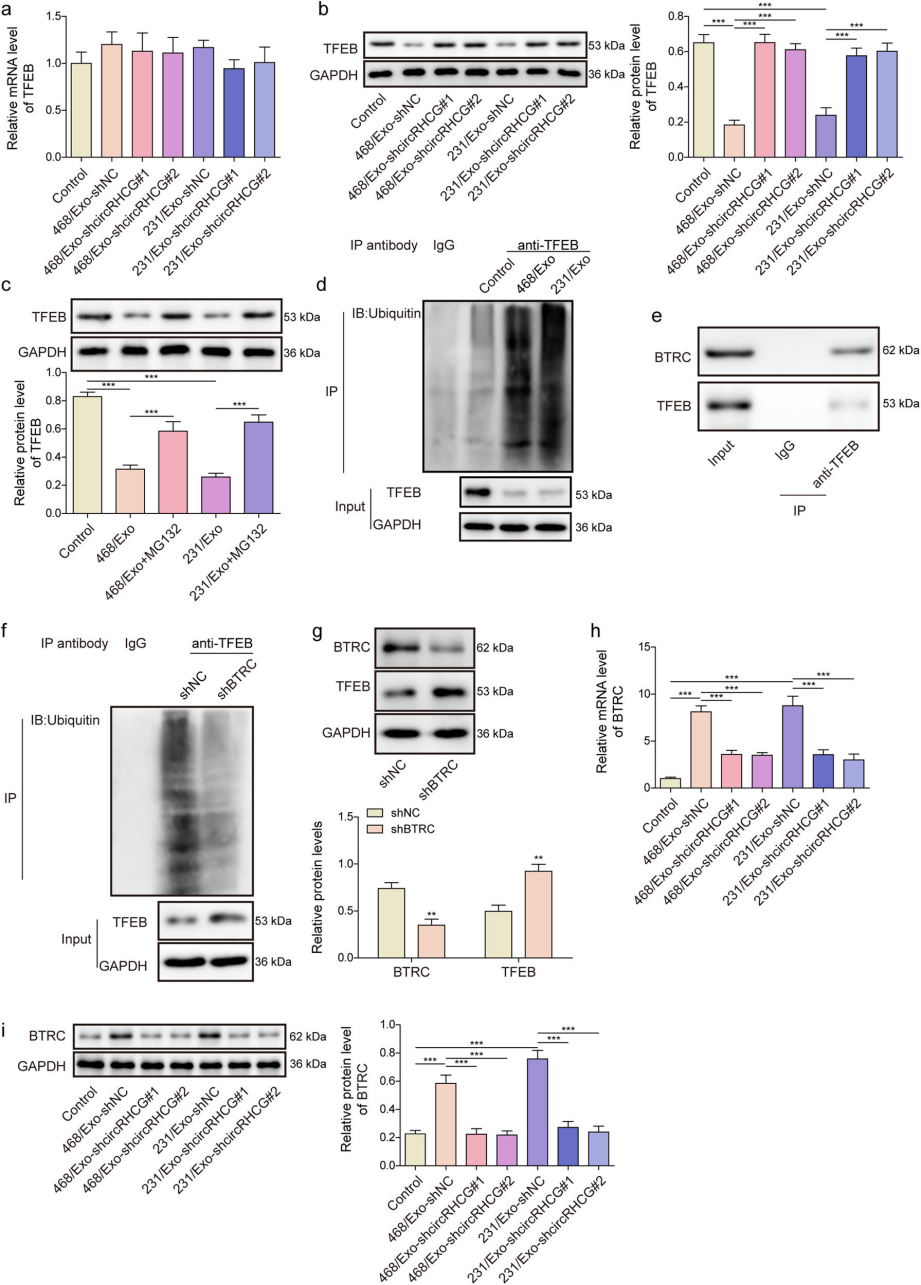
Exosomal circRHCG enhanced the ubiquitination and degradation of TFEB via upregulating BTRC. **a**, **b** The mRNA and protein levels of TFEB (*n* = 3). **c** Western blotting analysis of TFEB in THP-1 cells treated with exosomes (*n* = 3). MG132 was used to inhibit proteasome. **d** Ubiquitination analysis of immunoprecipitated TFEB from THP-1 cells treated with exosomes. **e** BTRC was coimmunoprecipitated with TFEB. **f** Ubiquitination analysis of immunoprecipitated TFEB from THP-1 cells after BTRC knockdown. **g** Protein levels of BTRC and TFEB in THP-1 cells (*n* = 3). **h**, **i** The mRNA and protein levels of BTRC (*n* = 3). ***p < 0.001. Error bars indicate SD. All results are representative of or combined from at least three independent experiments.

As TFEB is a vital regulator of breast cancer-associated macrophages^20^, we analyzed TFEB expression in THP-1 cells. The mRNA level of TFEB was unaffected, whereas MDA-MB-468 and MDA-MB-231 -derived exosomes reduced the protein level of TFEB, and exosomal knockdown of circRHCG abrogated the suppressive effect (Fig. 4a, b). Moreover, MG132 treatment also reversed exosome-mediated suppression of TFEB at protein level, indicating that exosomal circRHCG might regulate TFEB expression through the ubiquitin-proteasome mechanism (Fig. 4c). Furthermore, we found that MDA-MB-468 and MDA-MB-231 -derived exosomes markedly enhanced the ubiquitination level of TFEB (Fig. 4d). Co-immunoprecipitation assays showed that BTRC could be immunoprecipitated by an anti-TFEB antibody, suggesting the direct interaction between BTRC and TFEB (Fig. 4e). We determined the levels of ubiquitinated-TFEB and its protein expression after BTRC knockdown in THP-1 cells. Knockdown of BTRC significantly reduced the ubiquitination of TFEB and enhanced TFEB protein expression in THP-1 cells (Fig. 4f, g). MDA-MB-468 and MDA-MB-231-derived exosomes promoted BTRC expression in THP-1 cells, whereas circrRHCG-silencing exosomes did not have this effect (Fig. 4h, i). Our data demonstrated that exosomal circRHCG promoted BTRC expression and subsequently enhanced the ubiquitination and degradation of TFEB in THP-1 cells.

### CircRHCG stabilized BTRC mRNA via binding FUS

**Fig. 5:**
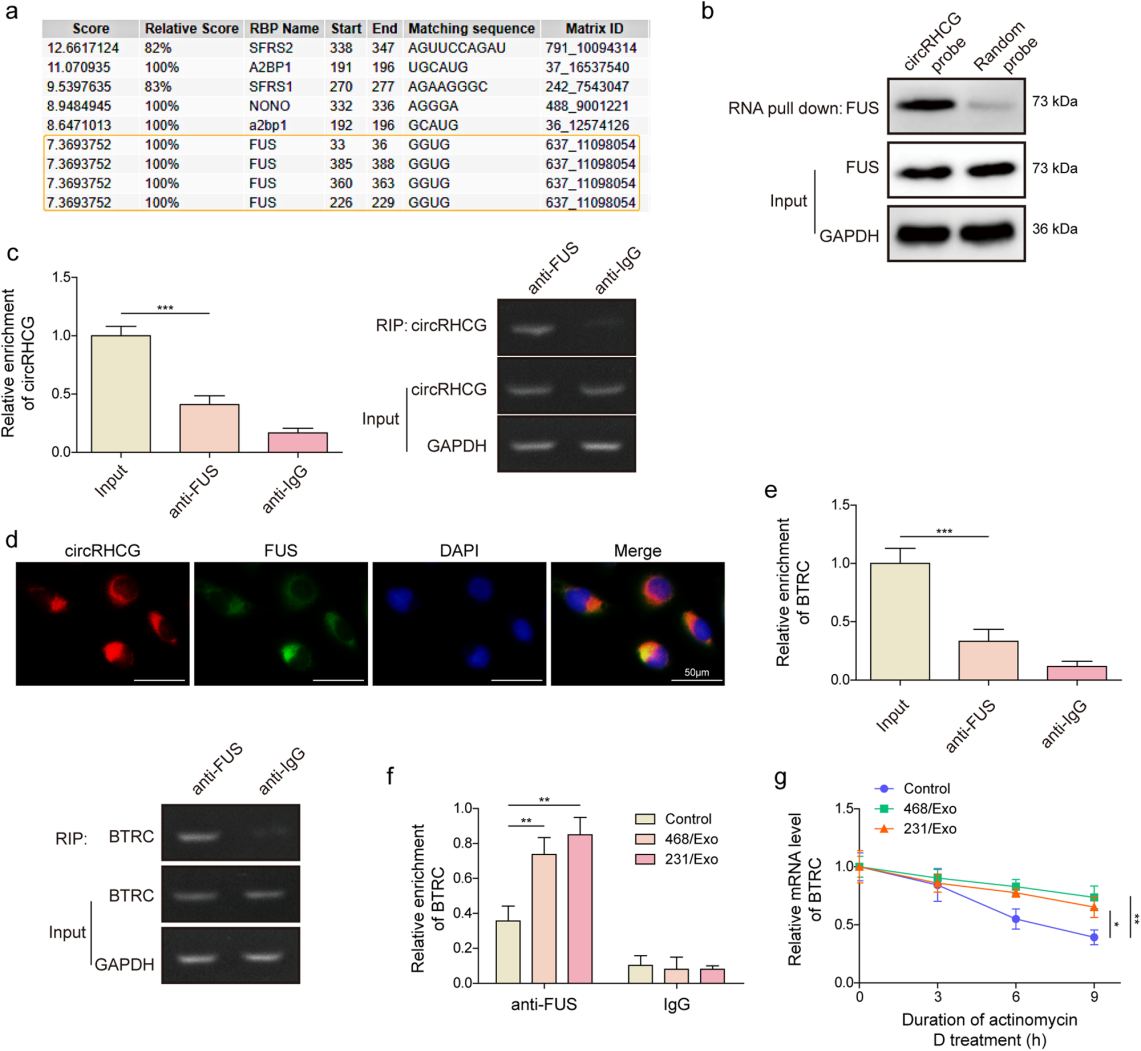
CircRHCG stabilized BTRC mRNA via binding FUS. **a** A binding site for FUS in circRHCG. **b** FUS was pulled down by the circRHCG probe, not by the random probe. **c** CircRHCG was enriched by the anti-FUS antibody (*n* = 3). **d** Colocalization of circRHCG (Red) and FUS (Green) was confirmed by combined FISH and IF staining. **e** BTRC mRNA was enriched by the anti- FUS antibody (*n* = 3). **f** The enrichment of BTRC mRNA by the anti-FUS antibody in THP-1 cells treated with exosomes (*n* = 3). **g** The mRNA levels of BTRC in THP-1 cells treated with exosomes in response to actinomycin D (*n* = 3). *p < 0.05, **p < 0.01 and ***p < 0.001. Error bars indicate SD. All results are representative of or combined from at least three independent experiments.

We predicted that circRHCG contained a potential binding site for FUS, an RNA-binding protein, through RBPDB (http://rbpdb.ccbr.utoronto.ca) (Fig. 5a). Indeed, FUS could be pulled down by the circRHCG probe, and circRHCG could be enriched by an anti-FUS antibody (Fig. 5b, c). In addition, FISH and immunofluorescence (IF) staining showed colocalization of FUS and circRHCG (Fig. 5d). These data suggested a direct interaction between circRHCG and FUS. Additionally, we found that BTRC mRNA could also be enriched by the anti-FUS antibody, and MDA-MB-468 and MDA-MB-231-derived exosomes further enhanced this enrichment (Fig. 5e, f). As FUS regulates the stability of target mRNAs^21,22^, we analyzed BTRC mRNA stability in response to actinomycin D treatment. MDA-MB-468 and MDA-MB-231-derived exosomes distinctly enhanced the stability of BTRC mRNA (Fig. 5g). These data suggested that exosomal circRHCG might stabilize BTRC mRNA via binding FUS and facilitating interaction between FUS and BTRC mRNA, thus naturally increasing BTRC expression.

### Knockdown of BTRC or overexpression of TFEB reversed exosomal circRHCG-induced M2 polarization

**Fig. 6:**
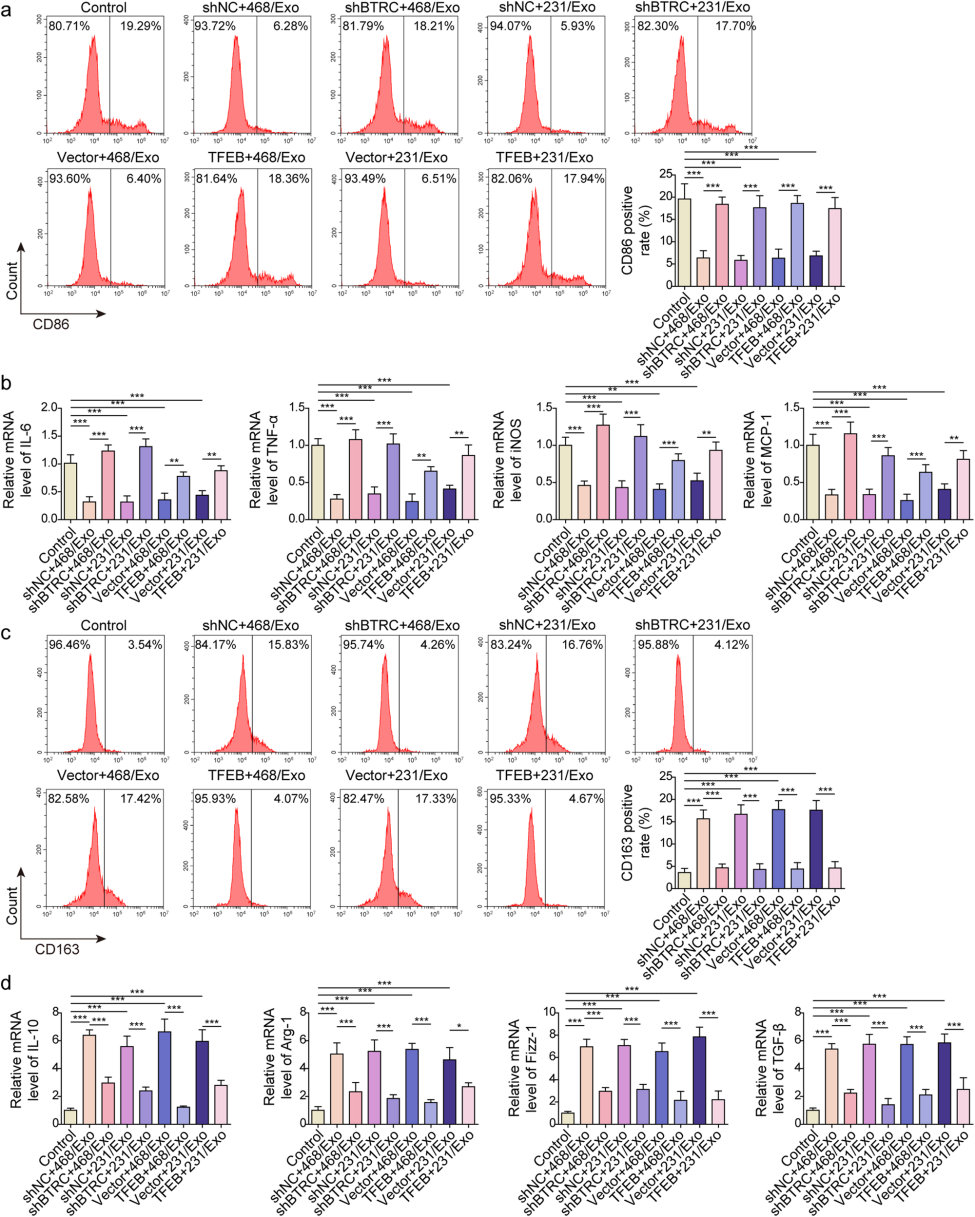
Knockdown of BTRC or overexpression of TFEB reversed exosomal circRHCG-induced M2 polarization. BTRC was knocked down or TFEB was overexpressed in THP-1 cells, and THP-1 cells were treated with PMA and subsequently co-cultured with MDA-MB-468 and MDA-MB-231 -derived exosomes. **a** Flow cytometry analysis of CD86 expression (*n* = 3). **b** RT-qPCR analysis of IL-6, TNF-α, iNOS, and MCP-1 (*n* = 3). **c** Flow cytometry analysis of CD163 expression (*n* = 3). **d** RT-qPCR analysis of IL-10, Arg-1, Fizz-1, and TGF-β (*n* = 3). *p < 0.05, **p < 0.01 and ***p < 0.001. Error bars indicate SD. All results are representative of or combined from at least three independent experiments.

To investigate whether exosomal circRHCG regulates M2 polarization through BTRC and TFEB, BTRC was knocked down and TFEB was overexpressed in THP-1 cells, and THP-1 cells were stimulated with PMA and subsequently co-cultured with MDA-MB-468 or MDA-MB-231-derived exosomes. MDA-MB-468 and MDA-MB-231 -derived exosome-mediated downregulation of CD86-positive M1 cells was reversed by knockdown of BTRC and overexpression of TFEB (Fig. 6a). Also, knockdown of BTRC and overexpression of TFEB reversed exosome-mediated suppression of IL-6, TNF-α, iNOS, and MCP-1 (Fig. 6b). Additionally, exosome-induced increase in CD163-positive M2 cells and the expression of IL-10, Arg-1, Fizz-1, and TGF-β was abrogated by knockdown of BTRC and overexpression of TFEB in THP-1 cells (Fig. 6c, d). Collectively, our findings suggested that exosomal circRHCG promoted M2 polarization through the BTRC/TFEB axis.

### Exosomal circRHCG promoted TNBC cell proliferation and metastasis by facilitating M2 polarization

**Fig. 7:**
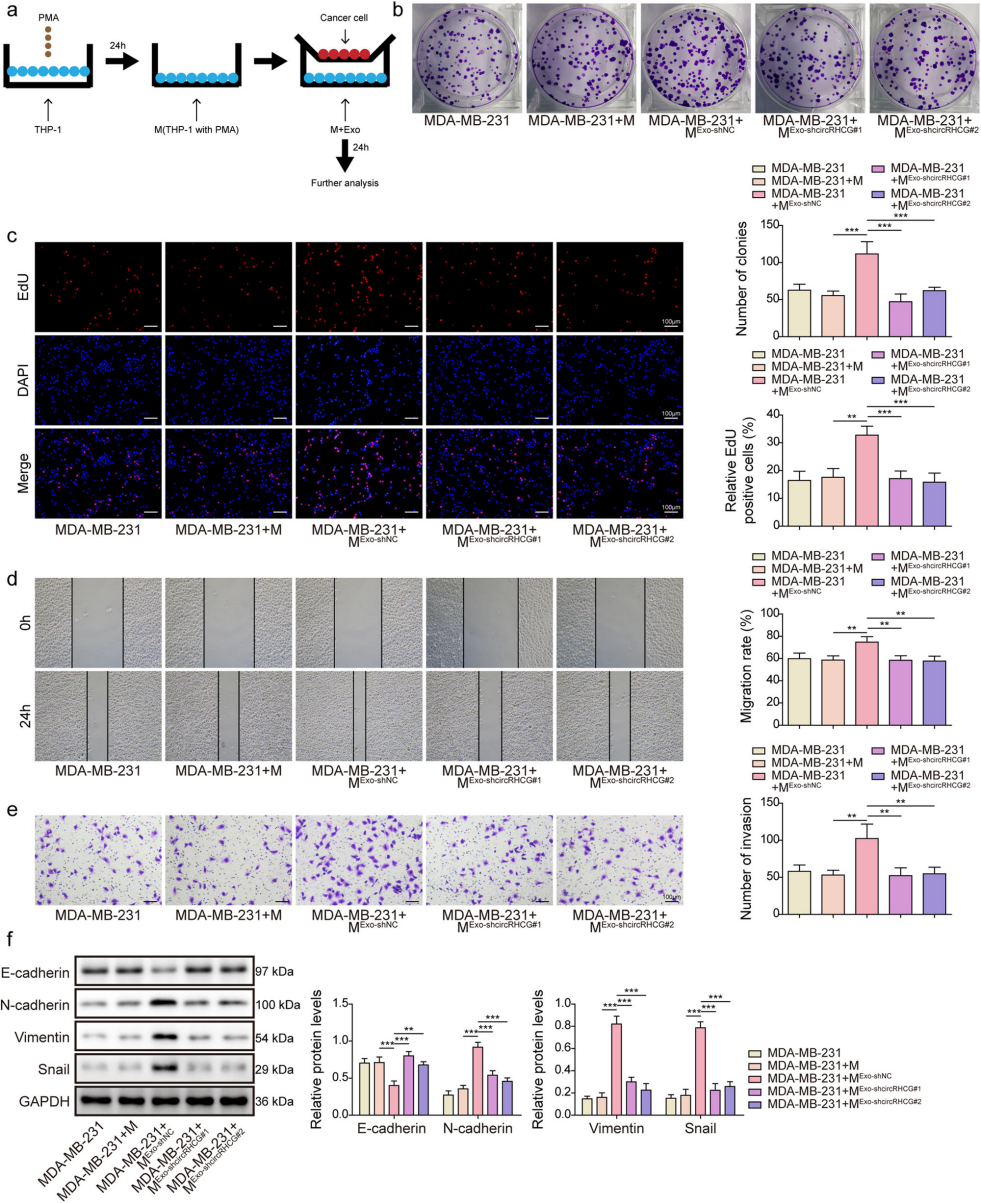
Exosomal circRHCG promoted TNBC cell proliferation and metastasis by facilitating M2 polarization. **a** THP-1 cells were stimulated with PMA, treated with exosomes and co-cultured with MDA-MB-231 cells for 24 h. **b** Colony formation analysis (*n* = 3). **c** EdU (Red) incorporation assays (*n* = 3). The nuclei were stained with DAPI (Blue). Scale bar, 100 µm. **d** Wound healing assays (*n* = 3). **e** Tanswell assays for analyzing cell invasion (*n* = 3). **f** Protein levels of E-cadherin, N-cadherin, Vimentin, Snail an GAPDH (*n* = 3). **p < 0.01 and ***p < 0.001. Error bars indicate SD. All results are representative of or combined from at least three independent experiments.

THP-1 cells were stimulated with PMA, treated with TNBC cell-derived normal or circRHCG-knockdown exosomes and co-cultured with MDA-MB-231 cells (Fig. 7a). THP-1 cells treated with normal exosomes significantly enhanced colony formation, proliferation, migration and invasion of MDA-MB-231 cells, but THP-1 cells treated with circRHCG-knockdown exosomes did not show these effects (Fig. 7b–e). In addition, normal exosome-treated THP-1 cells reduced E-cadherin expression and promoted the expression of N-cadherin, Vimentin, and Snail in MDA-MB-231 cells, but THP-1 cells treated with circRHCG-knockdown exosomes did not affect the expression of these EMT markers (Fig. 7f). Our data implied that exosomal circRHCG facilitated TNBC proliferation and metastasis via promoting M2 polarization.

### CircRHCG accelerated tumor growth, metastasis, and M2 polarization through the BTRC/TFEB axis

**Fig. 8:**
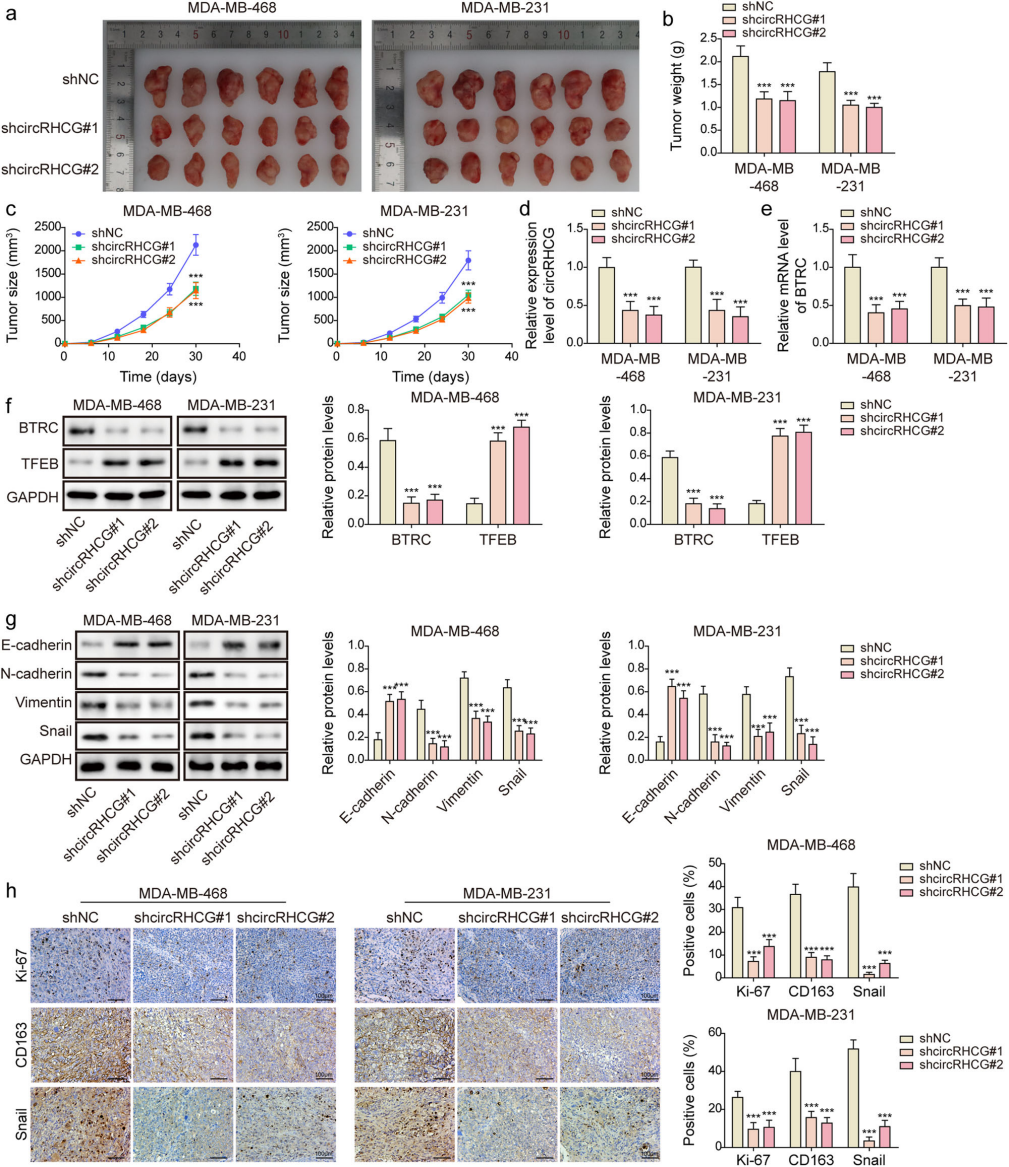
CircRHCG accelerated tumor growth, metastasis and M2 polarization through the BTRC/TFEB axis. **a** Images of excised tumors (*n* = 6 each group). **b**, **c** Tumor weight and volume (*n* = 6 each group). **d** RT-qPCR analysis of circRHCG in tumors (*n* = 6 each group). **e** The mRNA levels of BTRC in tumors (*n* = 6 each group). **f**, **g** The protein levels of BTRC, TFEB, E-cadherin, N-cadherin, Vimentin, Snail an GAPDH in tumors (*n* = 6 each group). **h** Immunohistochemistry staining of Ki-67, CD163, and Snail (*n* = 6 each group). ***p < 0.001. Error bars indicate SD.

**Fig. 9:**
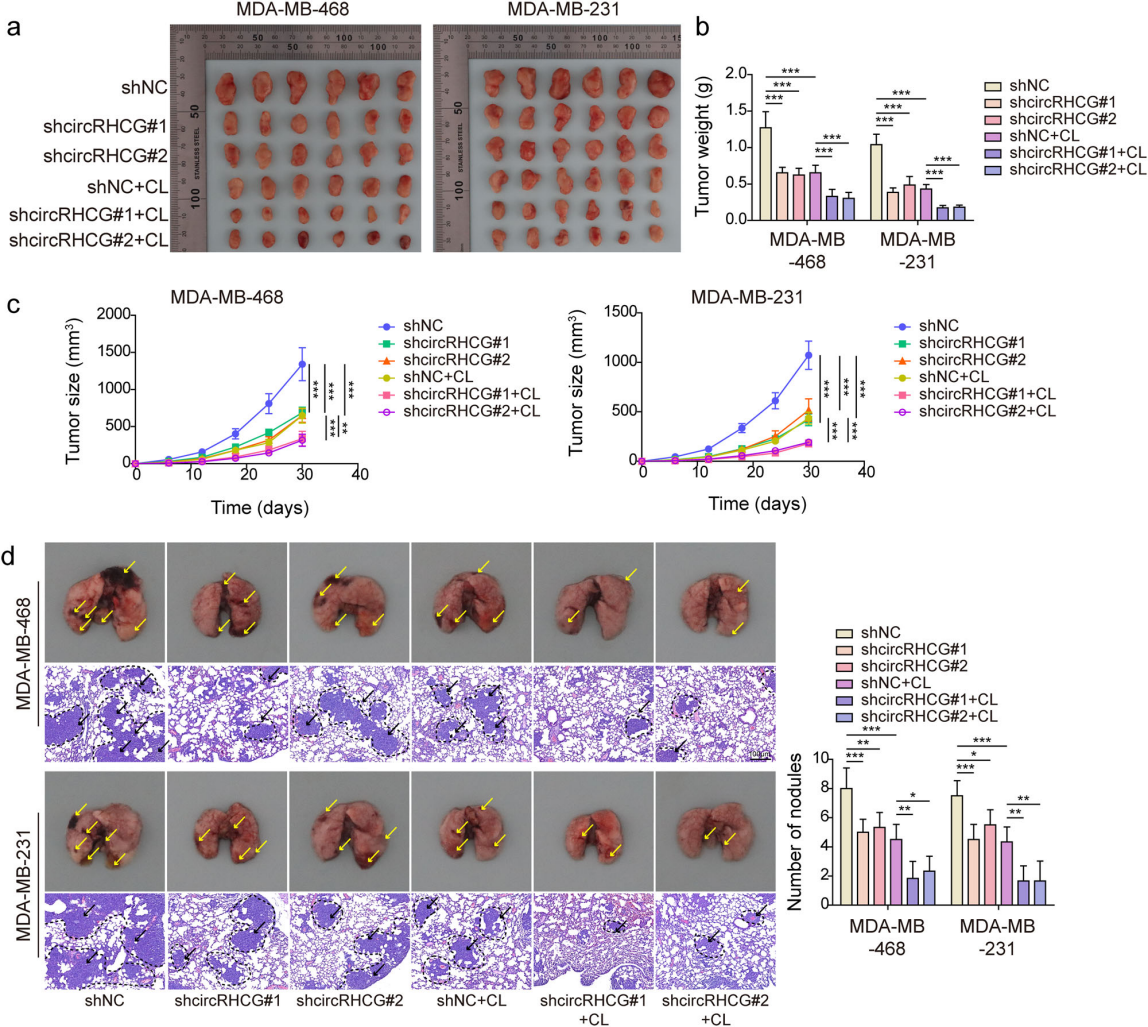
Knockdown of circRHCG inhibited tumor growth and metastasis. **a**–**c** MDA-MB-468 and MDA-MB-231 cells were orthotopically injected into the mammary fat pad, and tumors were imaged and weighed. Tumor volume was monitored (*n* = 6 each group). **d** MDA-MB-468 and MDA-MB-231 cells were injected into nude mice via tail vein. The lung was excised and imaged. Pulmonary metastasis was examined via H&E staining, and metastatic nodules were counted (*n* = 6 each group). *p < 0.05, **p < 0.01, and ***p < 0.001. Error bars indicate SD.

CircRHCG-knockdown MDA-MB-468 and MDA-MB-231 cells were subcutaneously injected into mice. Knockdown of circRHCG significantly suppressed subcutaneous tumor growth (Fig. 8a). Moreover, the tumor weight and volume were reduced by knockdown of circRHCG (Fig. 8b, c). Tumors formed by circRHCH-knockdown TNBC cells showed decreased expression of circRHCG and BTRC (Fig. 8d, e). BTRC was downregulated but TFEB was upregulated in tumors by knockdown of circRHCG (Fig. 8f). In addition, knockdown of circRHCG enhanced E-cadherin expression and suppressed the expression of N-cadherin, Vimentin and Snail in tumors (Fig. 8g). Besides, knockdown of circRHCG inhibited the expression of Ki-67, CD163 and Snail (Fig. 8h). To further evaluate circRHCG-silencing-mediated suppression of tumor growth and metastasis, in vivo xenograft mouse models were established by orthotopic mammary fat pad and tail vein injection of circRHCG-knockdown MDA-MB-468 and MDA-MB-231 cells. Clodronate liposome was used to deplete macrophages in vivo via intraperitoneal injection. Knockdown of circRHCG or macrophage depletion markedly reduced tumor size, weight, and volume, and circRHCG silencing-mediated anti-tumor activity was further strengthened by macrophage depletion via clodronate liposome administration (Fig. 9a–c). In addition, pulmonary metastasis was reduced by circRHCG knockdown or macrophage depletion, and macrophage depletion further suppressed the metastasis in tumors formed by circRHCG-silencing cells (Fig. 9d). Collectively, these observations suggested that circRHCG contributed to tumor progression and M2 polarization through the BTRC/TFEB axis in vivo.

## Discussion

**Fig. 10:**
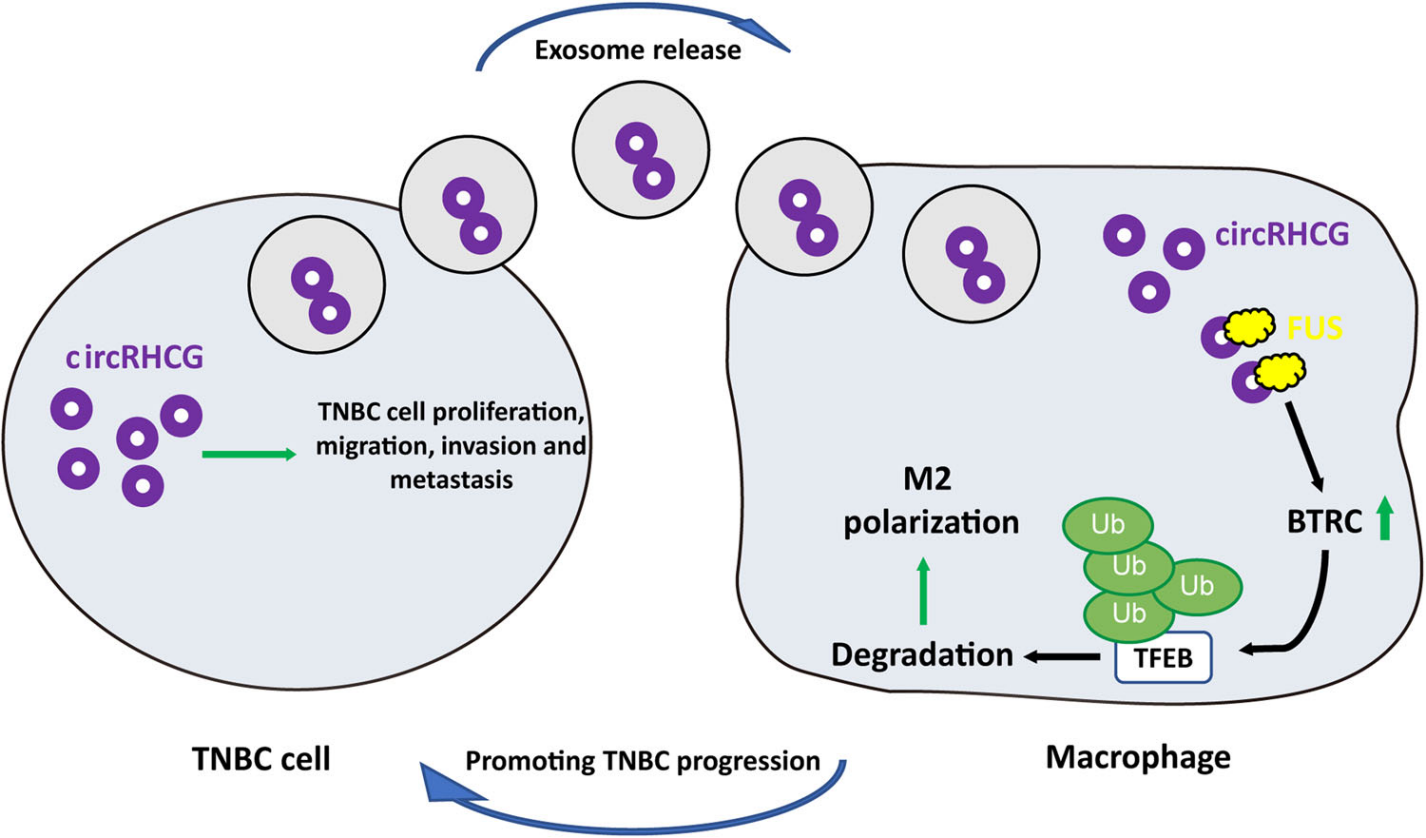
The schematic diagram of exosomal circRHCG-mediated regulation of M2 polarization and tumor progression in TNBC. Exosomal circRHCG binds to FUS and stabilizes BTRC mRNA to promote TFEB ubiquitination and degradation, thereby promoting TNBC progression by facilitating M2 polarization.

Approximately 2,000,000 new cases of breast cancer a year is reported, and TNBC accounts for about 15%^23,24^. TNBC more commonly affects women aged under 40 and shows lower 5-year survival rate (77%) than other types (93%)^25,26^. Besides survival rate, typically, TNBC patients have bad prognosis, early recurrence, and high risk of distant metastasis that causes great difficulty for treatment^27^. Thus, exploring the mechanisms underlying the metastasis of TNBC is vital for seeking therapies for TNBC. In this study, we found high abundance of circRHCG in TNBC-derived exosomes, and exosomal circRHCG promoted M2 polarization via enhancing BTRC-dependent ubiquitination and degradation of TFEB, thus facilitating tumor cell metastasis and accelerating TNBC progression (Fig. 10). Collectively, circRHCG accelerated TNBC progression via endogenously regulating the behaviors of TNBC cells and exosomal delivery to shape tumor microenvironment.

Exosomes are key regulators in intercellular communication via delivering cellular components. Exosomes secreted by cancer cells can be internalized by various cells, such as tumor cells^28^, fibroblasts^29^, and leukocytes^30^, which modulates the activity of recipient cells and shapes the tumor microenvironment that regulates tumor progression. In particular, accumulated evidence has shown that tumor cell-derived exosomes can educate macrophages into M2 tumor-associated macrophages, accelerating angiogenesis and metastasis^31^. Piao et al. reported that TNBC-derived exosomes induced M2 polarization and thus promoted tumor growth and metastasis^32^. Consistently, we found that exosomes released by TNBC cells facilitated M2 polarization and enhanced TNBC cell proliferation and metastasis. Owing to low immunogenicity and great biocompatibility, exosomes show great potential as a drug delivery system for tumor intervention^33^. Extracellular vesicles from TNBC cells carrying CCL5 on their surface influenced the behaviors of resident macrophages, rendering them pro-metastatic in nature, which ultimately led to tumorigenesis^34^. However, a recent study reported that TNBC-derived CSF-1-bearing extracellular vesicles promoted pro-inflammatory macrophages that was correlated with better clinical outcome^35^. These contrary results may attribute to various tumor microenvironment, but it needs further investigation.

CircRNAs are emerging as important regulators in cancers, including TNBC, exerting antitumor or oncogenic activities^36,37^. The abundance of circRNAs and their biological activities in carcinogenesis have been confirmed^38^. Li and colleagues found that exosomal circPDE8A released by tumor cells enhanced invasive growth in pancreatic cancer^39^. However, the roles of exosomal circRNAs in TNBC are largely unknown. In a previous study, researchers demonstrated the abundance of circRHCG (hsa_circ_ 0104852) in exosomes from MDA-MB-231 cells and breast cancer patients^17^, but its roles in TNBC has not been investigated. Here, we validated the presence of circRHCG in TNBC cell and patient-derived exosomes and found the association between exosomal circRHCG and TNBC patient survival. Furthermore, we demonstrated that TNBC cell-derived exosomal circRHCG promoted M2 polarization to enhance TNBC cell growth and metastasis, identifying an oncogenic activity of exosomal circRHCG in TNBC.

CircRNAs exert functions through various mechanisms including sponging miRNAs, interacting with RNA binding proteins and modulating gene transcription^40^. Specially, we found a binding site for the RNA-binding protein FUS and confirmed the interaction between circRHCG and FUS. FUS is a key regulator in maintaining RNA stability. FUS maintains GluA1 mRNA stability by binding it and controlling the length of poly (A) tail^21^. Consistently, we found that FUS interacted with BTRC mRNA and enhanced the stability of BTRC mRNA, naturally upregulating BTRC.

TFEB is an important regulator of macrophage polarization in the tumor microenvironment. Macrophages with reduced TFEB displaying M2 macrophage characteristics significantly accelerated tumor growth and angiogenesis^41^. Herein, we observed reduced expression of TFEB in macrophages after TNBC cell-derived exosome treatment, and overexpression of TFEB inhibited M2 polarization, suggesting that exosomal circRHCG-mediated regulation of M2 polarization was dependent on TFEB. However, the mechanism by which knockdown of TFEB enhanced M2 polarization in TNBC is unknown, which needs further investigation. During exploring the mechanism underlying exosomal circRHCG-mediated reduction of TFEB in macrophages, we found that TFEB were reduced at protein levels, but not mRNA levels, indicating circRHCG-mediated posttranslational modification of TFEB. Furthermore, increased BTRC expression was observed. It has been demonstrated that BTRC is pivotal for ubiquitination and degradation of various targets, such as phosphorylated inhibitor of NF-κB α isoform (IκBα), GLI family zinc finger 2 (Gli2) and β-catenin^42–44^. In consistence, we demonstrated that BTRC interacted with TFEB to promote its ubiquitination and degradation.

Taken together, we demonstrated that exosomal circRHCG from TNBC cells promoted TNBC progression via facilitating M2 polarization through BTRC-dependent ubiquitination and degradation of TFEB. Exosome-derived circRHCG accelerated TNBC invasion and metastasis, implying its potential as a therapeutic target for TNBC treatment and diagnostic and prognostic biomarker.

## Methods

### Clinical specimens

Blood samples were collected from forty patients diagnosed with TNBC and forty healthy volunteers at the First Affiliated Hospital of Nanjing Medical University used for exosome isolation. Patients and volunteers provided written consent. All clinical studies complied with relevant ethical regulations including the Declaration of Helsinki and were approved by the Ethics Committee of the First Affiliated Hospital of Nanjing Medical University (Ethics code 2022-SR-520).

### Cell culture and treatment

Human TNBC cell lines (MDA-MB-231, BT-549, MDA-MB-468, SUM-159 and MDA-MB-453) and non-tumorigenic epithelial MCF-10A cells, as well as non-TNBC cell lines (MDA-MB-415, MCF-7, T47D, BT474, and Skbr-3) were provided by Cell Bank of Chinese Academy of Sciences (Shanghai, China), and maintained in Dulbecco’s modified eagle medium (DMEM) Complete Medium (Merck, St. Louis, MO, USA). THP-1 cells (ATCC, Manassas, VA, USA) were maintained in Roswell Park Memorial Institute (RPMI) 1640 medium supplemented with 12% fetal bovine serum (FBS, Gibco, Waltham, MA, USA). The cell lines were authenticated by STR DNA profiling analysis, and were detected to be free of mycoplasma contamination. For macrophage polarization, THP-1 cells were stimulated with phorbol 12-myristate 13-acetate (PMA, Merck) at 100 ng/mL for 24 h and subsequently incubated with TNBC cell-derived exosomes (5 µg/mL) for 48 h. For co-culture of polarized THP-1 cells and TNBC cells, TNBC cells were added into transwell inserts for adherence, and the inserts were placed to the top of polarized THP-1 cells. GW4869 (20 μM, Selleck, Shanghai, China) was used for inhibition of exosome generation. For MG132 treatment, THP-1 cells were treated with MG132 (10 μM, Selleck) for 4 h.

### Isolation and characterization of exosomes

Whole blood was collected and allowed to clot, and serum was collected after centrifugation for exosome extraction with Total Exosome Isolation Reagent (from serum, Thermo Fisher Scientific, Waltham, MA, USA). TNBC cells (3 × 10^6^) were seeded and maintained for 24 hours, and the medium was replaced with fresh exosome-depleted medium. After 48 hours, the medium was harvested for exosome isolation using Total Exosome Isolation Reagent (from cell culture media, Thermo Fisher Scientific). The morphology, size, and concentration of exosomes were examined by transmission electron microscopy (TEM) and NanoSight NS300 (Malvern, UK), respectively. The expression of exosome markers including CD63, CD9, tumor susceptibility gene 101 (TSG101) and heat shock protein 70 (HSP70) and negative markers including Calnexin and GM130 was examined by Western blotting.

### Internalization of exosomes

Exosomes were stained with PKH-26 (2 µM, Sigma, St. Louis, MO, USA) for 30 min and washed twice in PBS to remove excessive PKH-26 dye. Subsequently, THP-1 cells were co-cultured with PKH-26-labeled exosomes or an equal volume of the PKH26-PBS control for 24 h and stained with DAPI (1 µg/mL, Beyotime, Shanghai, China) prior to examination under a fluorescence microscope. For co-staining of circRHCG and exosomes, Cy3-labeled circRHCG was transfected into MDA-MB-468 and MDA-MB-231, and exosomes were extracted from culture supernatants and labeled with PKH67 (Sigma) at 2 µM for 30 min. THP-1 cells were incubated with exosomes for 24 h and stained with DAPI (1 µg/mL, Beyotime) prior to examination under a fluorescence microscope.

### Characterization of circRHCG

RNA was isolated and treated with RNase R (5 U/μg, Abcam, Cambridge, UK) or mock for 1 h at 37 °C. The abundance of Rh family C glycoprotein (RHCG) mRNA and circRHCG was detected with quantitative reverse transcription PCR (RT-qPCR). For inhibition of gene transcription, cells were treated with actinomycin D (Selleck) at 2 μg/mL for 0, 4, 8, 12, 16, and 20 h prior to RNA isolation, and RHCG mRNA and circRHCG was examined by RT-qPCR. Divergent primers were designed to amplify circRHCG, and the back-splicing site of circRHCG was confirmed by Sanger sequencing.

### Cell transfection

The coding region of TFEB was inserted into the pcDNA3.1 vector for TFEB overexpression. ShRNAs against circRHCG (shcircRHCG#1 and 2) and BTRC (shBTRC) and scramble shRNA controls (shNCs) were designed by RiboBio (Guangzhou, China). Cells were transfected with the TFEB-overexpressing construct, shcircRHCG, shBTRC, or shNC using Lipofectamine 3000 (Thermo Fisher Scientific). For stable transfection, shcircRHCG or shNC was cloned into the lentiviral vector pLKO.1, and lentiviral particles were packaged in 293T cells (ATCC) for stably silencing of circRHCG.

### Fluorescence in situ hybridization and immunofluorescence staining

Combined FISH/IF staining was performed as previously described with modification^45^. In brief, cells were seeded on coverslips and fixed in 3.7% paraformaldehyde solution and permeabilized with 0.5% Triton X-100 solution. Subsequently, cells were digested with proteinase K at 10 µg/µL for 20 min and pre-hybridized for 1 h. Cells were hybridized with Cyanine 3 (Cy3)-conjugated circRHCG, U6 or 18S rRNA probes at 50 nM for 12 h. For IF staining, after probe hybridization, cells were incubated with a rabbit anti-fused in sarcoma (FUS, 1:200, ab243880, clone: BLR023E, Abcam) overnight. Cells were incubated with an Alexa Fluor 488-conjugated secondary antibody (ab150077, Abcam) for 1 h and mounted with antifade mountant with 4’,6-diamidino-2-phenylindole (DAPI, Thermo Fisher Scientific).

### Colony formation

TNBC cells (1 × 10^3^) were treated and maintained for 14 d in serum-free DMEM in a cell incubator. Cell colonies were fixed and stained with crystal violet (1%, Sigma). Subsequently, cell colonies were rinsed and imaged.

### EdU incorporation

EdU incorporation was performed using the Click-IT EdU kit (Thermo Fisher Scientific). Briefly, cells were treated and incubated in fresh culture medium containing 10 µM of EdU for 2 h. Subsequently, cells were fixed, permeabilized and incubated in Click-iT reaction cocktail solution for 30 min. After wash, cells were mounted with antifade mountant with DAPI (Thermo Fisher Scientific).

### Wound healing assay

TNBC cells were seeded in 6-well plates in completed DMEM medium and cultured to a monolayer with 90% confluence. A wound of 1 mm was scratched using a pipette tip. Subsequently, cells were washed in PBS and maintained in serum-free DMEM medium for 24 h in a cell incubator. Wound healing was evaluated with an Olympus BX51 microscope (Tokyo, Japan).

### Transwell assay

Transwell chambers (BD, Franklin Lakes, NJ, USA) were applied for evaluating cell invasion. Matrix gel was pre-coated on the upper chamber. After indicated treatment, cells were seeded and incubated for 24 h. Cells which invaded into the lower chamber were fixed, stained with crystal violet (1%, Sigma) and imaged under an Olympus BX51 microscope.

### Flow cytometry analysis of CD86 and CD163 expression

After treatment, THP-1 cells were washed and detached using Trypsin (Thermo Fisher Scientific). Subsequently, 1 × 10^6^ cells were resuspended in 100 µL of phosphate-buffered saline (PBS)/2% bovine serum albumin (BSA) solution and incubated with a CD86-PE (5 µL, 12-0869-42, clone: IT2.2, Thermo Fisher Scientific) or CD163-PE (5 µL, 333605, clone: GHI/61, BioLegend, San Diego, CA, USA) antibody for 40 min. After rinse twice, cells were resuspended in 500 µL of PBS/2% BSA solution. The cells were detected Beckman cytoflex flow cytometry (Beckman, USA). The Cytexpert Software was applied for the flow cytometry data analysis. The gating strategy for flow cytometry analysis in Figs. 3 and 6 was provided in Supplementary Figure no. 2.

### RNA pull-down assay

THP-1 cells were lysed, and cell lysates were collected. Biotinylated circRHCG and random probes (RiboBio) were mixed thoroughly with cell lysates and incubated overnight at 4 °C. Streptavidin magnetic beads (Thermo Fisher Scientific) were added into samples and incubated for 2 h with gentle rotation. Subsequently, protein was recovered from the complexes pulled down by probes and applied for Western blotting analysis of FUS using a rabbit anti-FUS antibody (1:500, ab243880, clone: BLR023E, Abcam).

### Co-immunoprecipitation

THP-1 cells were lysed, and cell lysates were collected. An anti-TFEB antibody (5 µg, ab264421, clone: BLR070G, Abcam) or normal Immunoglobulin G (IgG, 5 µg, Abcam) was mixed with cell lysates and incubated for 16 h. Samples were mixed with Protein A/G magnetic beads (Sigma) and incubated for 1 h with gentle rotation. Protein was recovered and applied for Western blotting analysis of BTRC abundance or TFEB ubiquitination using a rabbit anti-BTRC antibody (1:100, ab233739, clone: 3D5E6, Abcam) or anti-ubiquitin antibody (1:500, ab134953, clone: EPR8830, Abcam).

### RNA immunoprecipitation

THP-1 cells were lysed, and cell lysates were collected. An anti-FUS antibody (5 µg, ab243880, clone: BLR023E, Abcam) or normal IgG (5 µg, Abcam) was mixed with cell lysates and incubated overnight. Protein A/G magnetic beads (Sigma) were added into samples and incubated for additional 1 h. Subsequently, RNA was recovered, and the abundance of circRHCG and BTRC mRNA was examined with RT-qPCR.

### In vivo xenograft experiments

BALB/c nude mice (6-week-old; Female) were provided by SJA Laboratory Animal Co., Ltd (Hunan, China) and randomly divided into 3 groups (*n* = 6 per group): shNC, shcircRHCG#1 and shcircRHCG#2. TNBC cells (3 × 10^6^) stably transfected with shNC, shcircRHCG#1 or shcircRHCG#2 were subcutaneously injected into the left flanks. The tumor volume was measured every 6 days and calculated with the formula length × width^2^/2. After 30 days, mice were sacrificed, and tumors were excised for imaging and weighing. For orthotopic mammary fat pad injection, mice were blindly divided into 5 groups (*n* = 6 per group): shNC, shcircRHCG#1, shcircRHCG#2, shcircRHCG#1 + clodronate liposome (CL, depletion of macrophages) and shcircRHCG#2 + CL. TNBC cells (5 × 10^5^) were suspended in 100 μL of the mixture of PBS and Matrigel (1:1) and injected into the mammary fat pad. For CL administration, mice were intravenously injected with CL (10 μL/g/week). The tumor volume was measured every 5 days and calculated with the formula length × width^2^/2. After 30 days, tumors were excised, imaged, and weighed. To evaluate pulmonary metastasis, mice were blindly divided into 5 groups (*n* = 6 per group): shNC, shcircRHCG#1, shcircRHCG#2, shcircRHCG#1 + CL and shcircRHCG#2 + CL. TNBC cells (1 × 10^5^) were suspended in 100 μL of PBS and injected into mice via tail vein, and CL (10 μL/g/week) was intravenously injected into mice. After 21 days, the lungs were excised and imaged. Standard hematoxylin and eosin (H&E) staining was performed to evaluate the formation of metastatic nodules. The investigator was blinded to the group allocation during the experiment. All animal experimental procedures acquired official approval from the Institutional Animal Care and Use Committee of Nanjing Medical University (IACUC-2206005).

### Immunohistochemistry staining

Subcutaneous tumors were excised and fixed in 4% paraformaldehyde. Tumors were hydrated and embedded in paraffin, which were sliced into 5-µm sections. Sections were then deparaffinized and rehydrated, and antigen was retrieved in antigen retrieval buffer in a microwave. Sections were incubated with antibodies against Ki-67 (1:100, ab16667, clone: SP6, Abcam), CD163 (1:50, ab182422, clone: EPR19518, Abcam) and Snail (1:100, PA5-23482, Thermo Fisher Scientific) overnight. After wash, sections were incubated with a horseradish peroxidase (HRP)-conjugated secondary antibody (31460, Thermo Fisher Scientific) for 1 h, and signal was visualized with diaminobenzidine (DAB, Beyotime).

### RT-qPCR

Total RNA was isolated from exosomes, TNBC cells, THP-1 cells, and tumors using Trizol reagent (Thermo Fisher Scientific) and reversely transcribed into cDNA. The expression of circRHCG, RHCG, interleukin-6 (IL-6), tumor necrosis factor-α (TNF-α), inducible nitric oxide synthase (iNOS), monocyte chemoattractant protein-1 (MCP-1), IL-10, arginase-1 (Arg-1), found in inflammatory zone 1 (Fizz-1), transforming growth factor-β (TGF-β), TFEB and BTRC was examined using quantitative PCR with SYBR Green (TOYOBO, Tokyo, Japan) and normalized to glyceraldehyde-3-phosphate dehydrogenase (GAPDH). 2^−∆∆Ct^ method was applied for calculation. Primers were shown in Supplementary Table 1.

### Western blotting

Tumor tissues were homogenized in ice-cold lysis buffer. Protein was extracted from TNBC cells, THP-1 cells, exosomes and tumor homogenates using Protein Extraction Kit (Abcam) and quantified with bicinchoninic acid (BCA) kit (Bio-Rad, Hercules, CA, USA). 30 µg of protein was electrophoresed in 12% SDS-PAGE gel and transferred to polyvinylidene fluoride (PVDF) membranes (Bio-Rad). Membranes were blocked and incubated with rabbit antibodies against CD63 (1:1000, ab193349, clone: MX-49.129.5, Abcam), CD9 (1:1000, ab236630, clone: EPR23105-121, Abcam), TSG101 (1:2000, ab125011, clone: EPR7130(B), Abcam), HSP70 (1:500, ab2787, clone: 5A5, Abcam), Calnexin (1:1000, ab22595, Abcam), GM130 (1:1000, ab52649, clone: EP892Y, Abcam), E-cadherin (1:500, ab231303, clone: 4A2, Abcam), N-cadherin (1:1000, ab76011, clone: EPR1791-4, Abcam), Vimentin (1:500, ab92547, clone: EPR3776, Abcam), Snail (1:500, PA5-23482, Thermo Fisher Scientific), TFEB (1:1000, ab264421, clone: BLR070G, Abcam), BTRC (1:800, ab71753, Abcam) and GAPDH (1:5000, ab8245, clone: 6C5, Abcam) overnight. Membranes were rinsed and incubated with an HRP-conjugated secondary antibody. Enhanced chemiluminescence (ECL) substrate (Beyotime) was used to visualize bands. Antibodies were ordered from Abcam. Uncropped scans were supplied in Supplementary Figure no. 3.

### Enzyme-linked immunosorbent assay

The concentration of IL-6, TNF-α, iNOS, MCP-1, IL-10, Arg-1, Fizz-1, and TGF-β in the culture supernatants was determined with commercial enzyme-linked immunosorbent assay (ELISA) kits following the manuals. Human IL-6 (ab178013), TNF-alpha (ab285312), iNOS (ab253217), MCP-1 (ab179886), IL-10 (ab185986), TGF beta 1 (ab100647) ELISA kits were provided by Abcam. Arginase-1 Human ELISA Kit (BMS2216TEN) and Human Fizz-1 ELISA Kit (SEKH-0305) were obtained from Thermo Fisher Scientific and SOLARBIO (Beijing, China), respectively.

### Statistical analysis

All experiments were conducted with three replicates, and each experiment was performed in triplicate. The data was expressed as mean ± standard deviation (SD). A priori power analysis (G*Power software) was performed to estimate sample size required to generate 80% power for detecting a significant (*p* < 0.05) effect of treatment. The normality of data was assessed by the Shapiro-Wilk test. Considering a significance level of 5%, there were no significant deviations from the normality of all data (*p* > 0.05). The Student’s *t* test (two tailed) and one-way analysis of variance (ANOVA) were applied to analyze the variance of two and more groups, respectively. Patient survival was compared by the Kaplan Meier curve. *P* < 0.05 was statistically significant.

### Reporting summary

Further information on research design is available in the Nature Research Reporting Summary linked to this article.

### Supplementary information

Supplementary Information

Reporting Summary

## Data Availability

Data will be made available on request.
