# Supplementary material for: Exosomal circRHCG promotes breast cancer metastasis via facilitating M2 polarization through TFEB ubiquitination and degradation
Source: NPJ Precis Oncol. 2024 Jan 29;8:22. doi: 10.1038/s41698-024-00507-y (PMC10825185; doi:10.1038/s41698-024-00507-y)
Supplement: Supplementary file 1 — Supplementary Information [file 41698_2024_507_MOESM1_ESM.pdf]

Supplementary Information

Supplementary Figure no. 1. Proteins from exosomes and TNBC cells were stained with Ponceau.

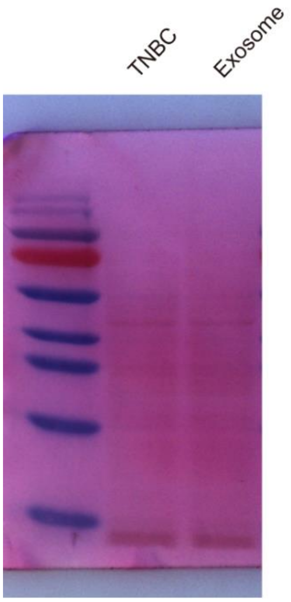

Supplementary Figure no. 2. The gating strategy for flow cytometry analysis in Figure 3 and 6 was provided.

Figure 3

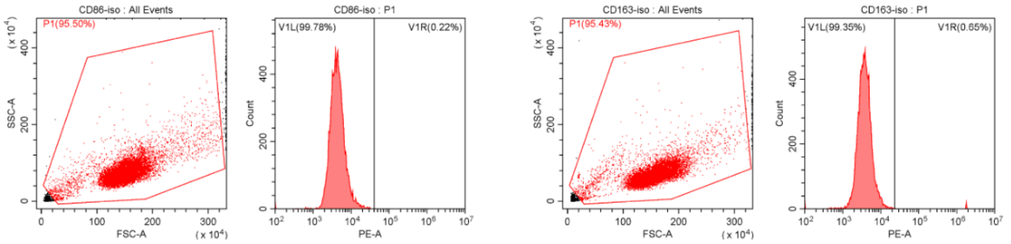

Figure 6

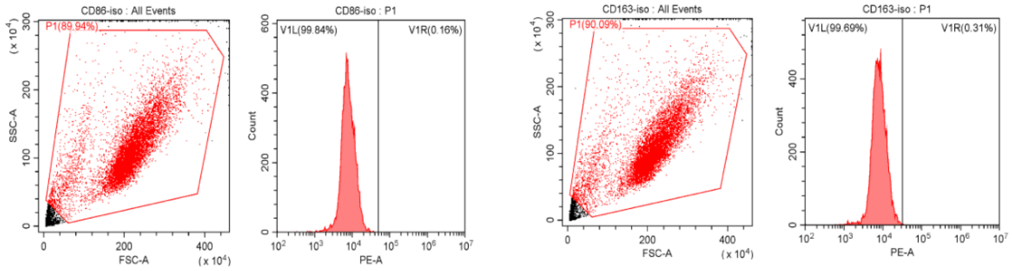

Supplementary Figure no. 3. Uncropped scans were provided.

Figure 1c

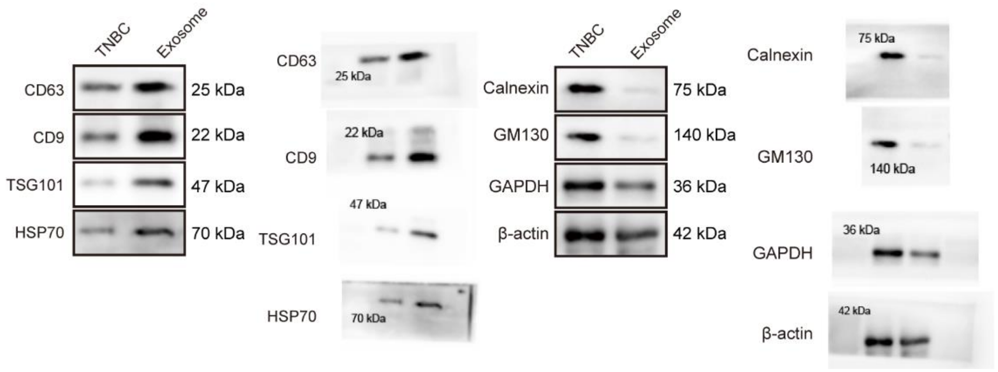

Figure 2f

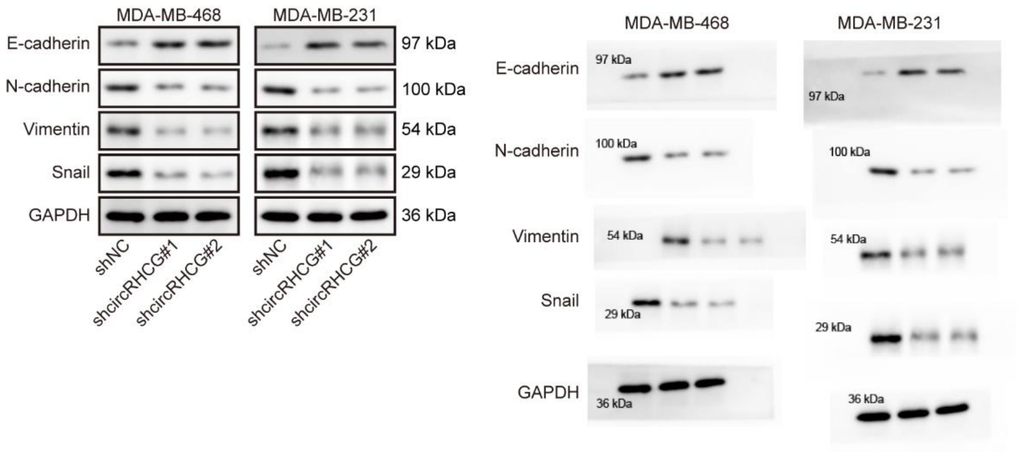

Figure 4b

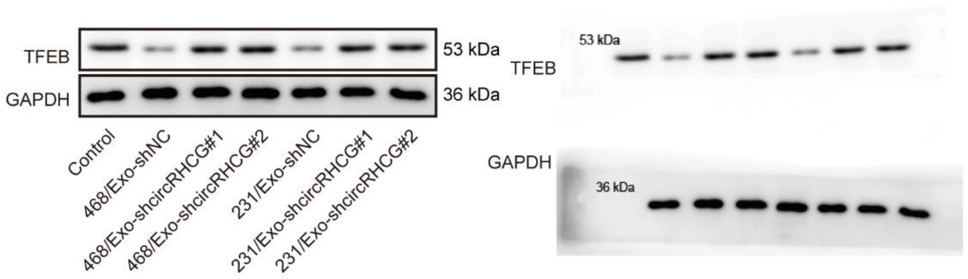

Figure 4c

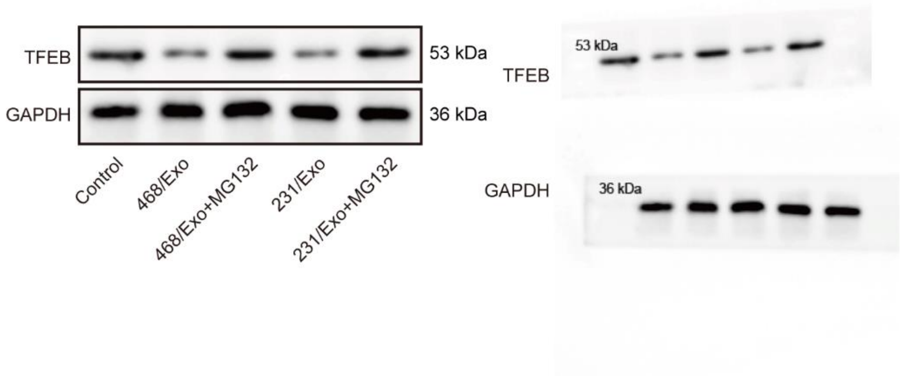

Figure 4d

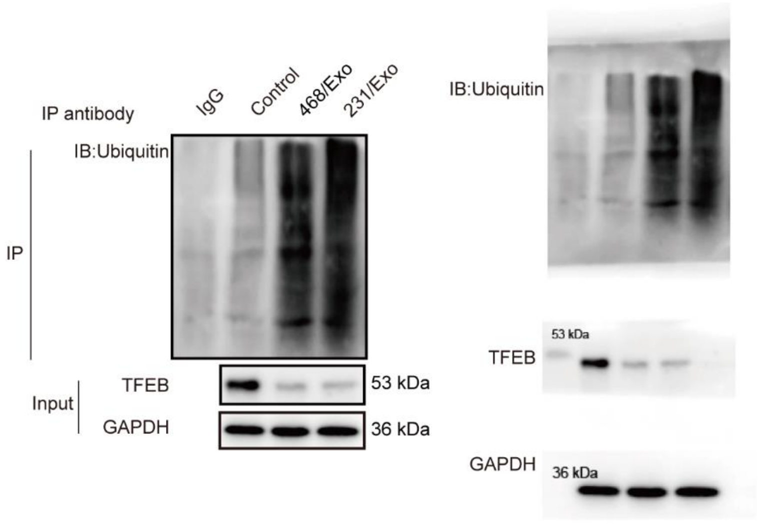

Figure 4e

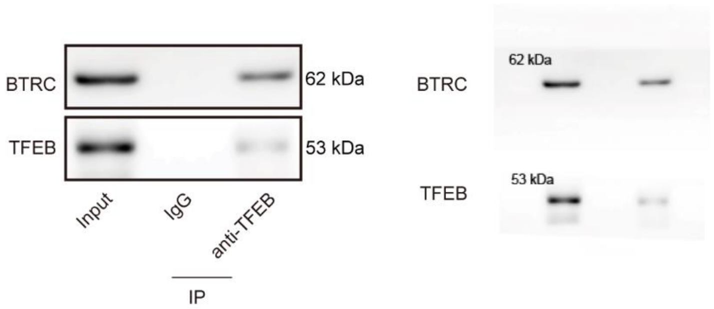

Figure 4f

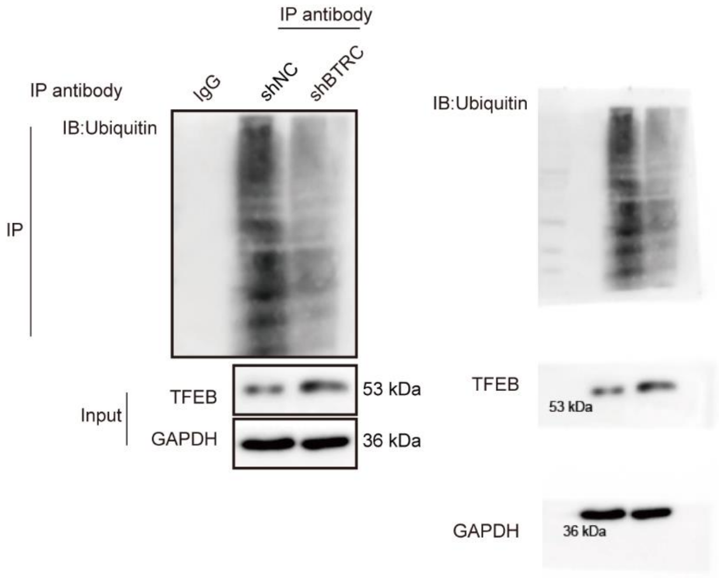

Figure 4g

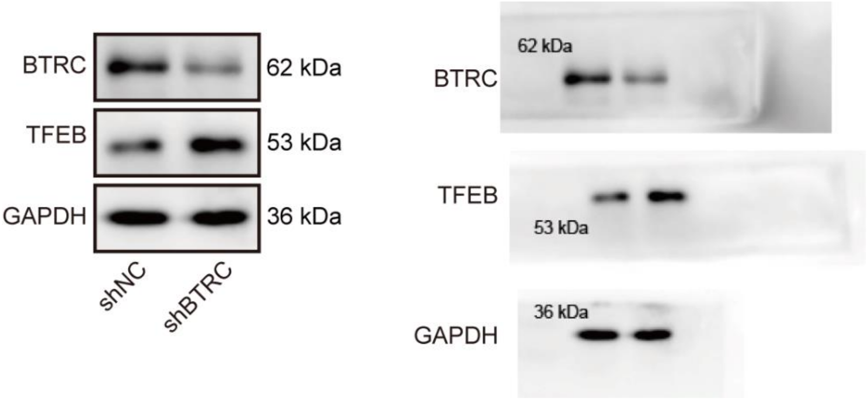

Figure 4i

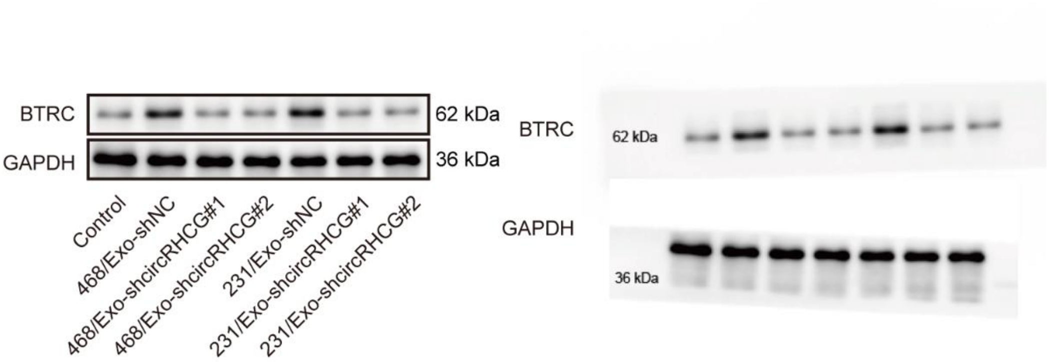

Figure 5b

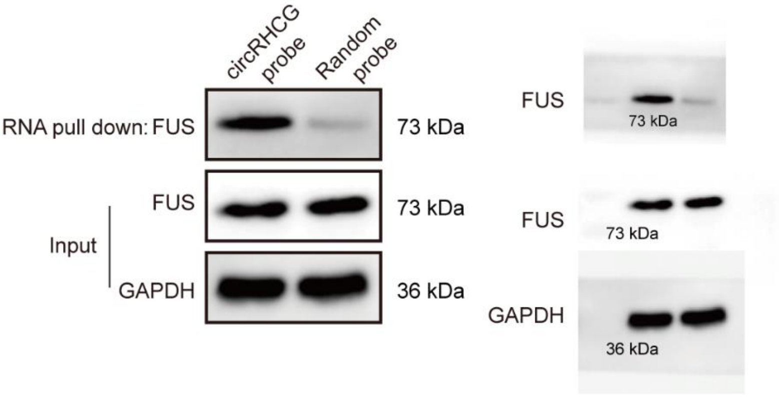

Figure 5c

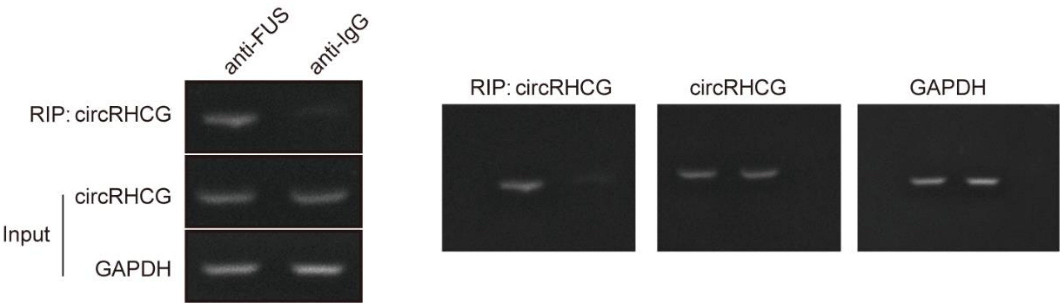

Figure 5e

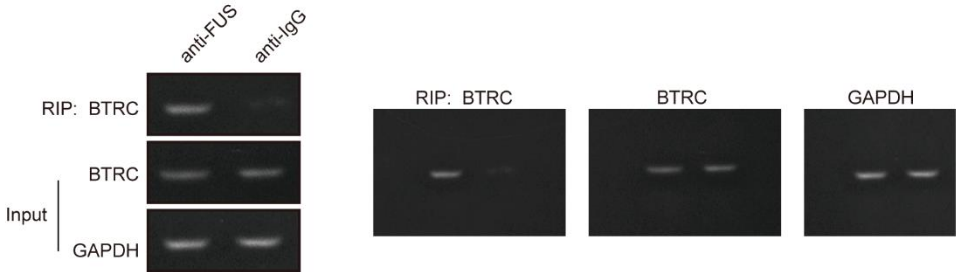

Figure 7f

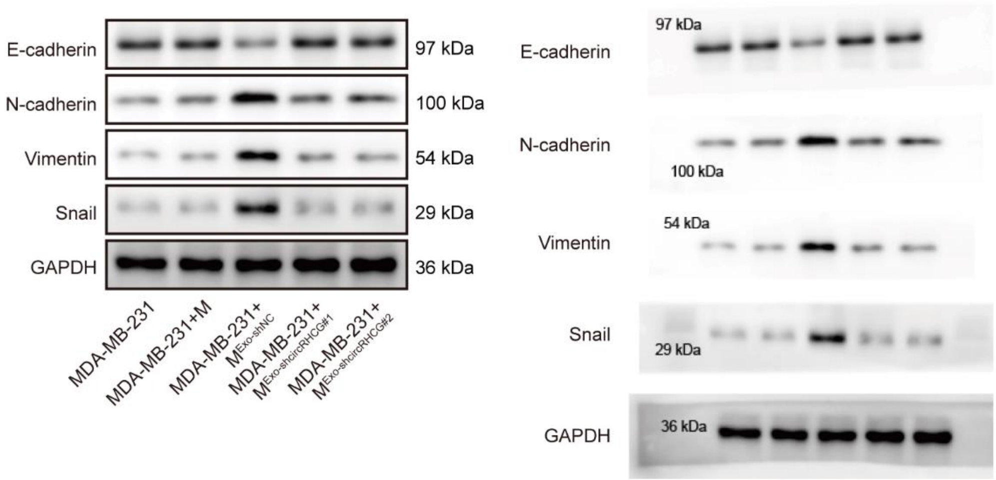

Figure 8f

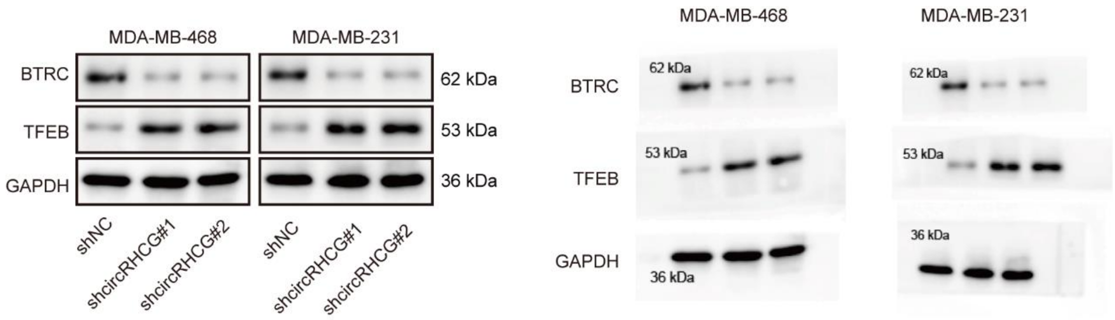

Figure 8g

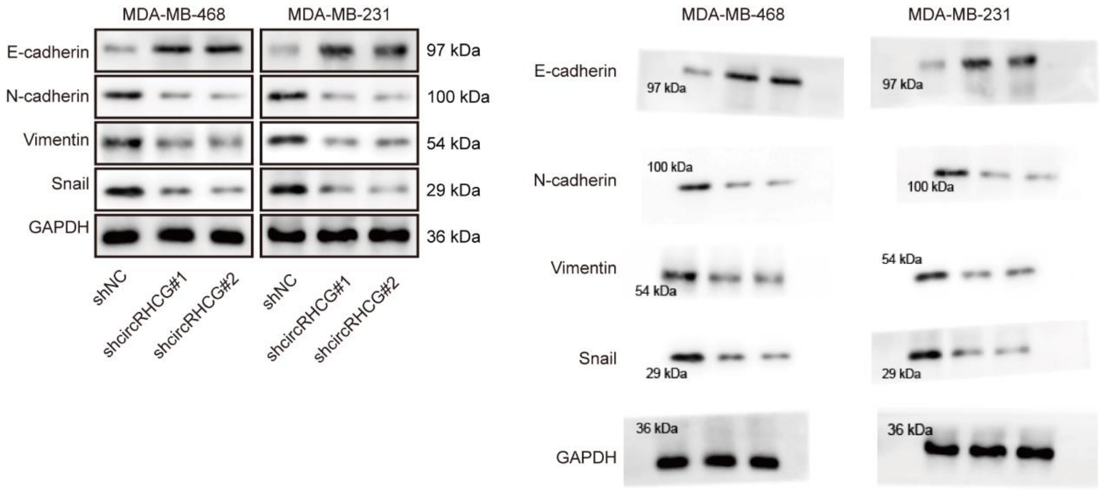

Supplementary Table 1 RT-qPCR primers in this study.

Genes	Primer sequences (5'-3')
circRHCG	Forward: 5’-GATGGGTGGCATCATTGTGG-3’<br>Reverse: 5’-GATGTGCACCTCCCAGTAGA-3’
RHCG	Forward: 5’-GATTTATGGTCTCTTGGTGACCCTG-3’<br>Reverse: 5’-CTAGCTAGGTCAGCACCAGCTC-3’
IL-6	Forward: 5’-ACTCACCTCTTCAGAACGAATTG-3’<br>Reverse: 5’-CCATCTTTGGAAGGTTCAGGTTG-3’
TNF-α	Forward: 5’-CCTCTCTCTAATCAGCCCTCTG-3’<br>Reverse: 5’-GAGGACCTGGGAGTAGATGAG-3’
iNOS	Forward: 5’-CAGCGGGATGACTTTCCAA-3’<br>Reverse: 5’-AGGCAAGATTTGGACCTGCA-3’
MCP-1	Forward: 5’-CAGCCAGATGCAATCAATGCC-3’<br>Reverse: 5’-TGGAATCCTGAACCCACTTCT-3’
IL-10	Forward: 5’-GCCTAACATGCTTCGAGATC-3’<br>Reverse: 5’-TGATGTCTGGGTCTTGGTTC-3’
Arg-1	Forward: 5’- GTGGAAACTTGCATGGACAAC -3’<br>Reverse: 5’- AATCCTGGCACATCGGGAATC -3’
Fizz-1	Forward: 5’-GCAAGAAGCTCTCGTGTGCTAG-3’<br>Reverse: 5’-AACATCCCACGAACCACAGCCA-3’
TGF-β	Forward: 5’-CCCAGCATCTGCAAAGCTC-3’<br>Reverse: 5’-GTCAATGTACAGCTGCCGCA -3’
TFEB	Forward: 5’-CCAGAAGCGAGAGCTCACAGAT-3’<br>Reverse: 5’-TGTGATTGTCTTTCTTCTGCCG-3’
BTRC	Forward: 5’-ACCAACATGGGCACATAAACTC-3’<br>Reverse: 5’-TGGCATCCAGGTATGACAGAAT-3’
GAPDH	Forward: 5’-ACCCACTCCTCCACCTTTGA-3’<br>Reverse: 5’-CTGTTGCTGTAGCCAAATTCGT-3’
